# The role of an amphiphilic helix and transmembrane region in the efficient acylation of the M2 protein from influenza virus

**DOI:** 10.1038/s41598-023-45945-z

**Published:** 2023-11-02

**Authors:** Xiaorong Meng, Clark Templeton, Cecilia Clementi, Michael Veit

**Affiliations:** 1 Institute of Virology, Veterinary Faculty, Freie Universität Berlin, Berlin, Germany; 2 Theoretical and Computational Biophysics, Department of Physics, Freie Universität Berlin, Berlin, Germany

**Keywords:** Computational biophysics, Post-translational modifications, Influenza virus, Biochemistry, Enzymes

## Abstract

Protein palmitoylation, a cellular process occurring at the membrane-cytosol interface, is orchestrated by members of the DHHC enzyme family and plays a pivotal role in regulating various cellular functions. The M2 protein of the influenza virus, which is acylated at a membrane-near amphiphilic helix serves as a model for studying the intricate signals governing acylation and its interaction with the cognate enzyme, DHHC20. We investigate it here using both experimental and computational assays. We report that altering the biophysical properties of the amphiphilic helix, particularly by shortening or disrupting it, results in a substantial reduction in M2 palmitoylation, but does not entirely abolish the process. Intriguingly, DHHC20 exhibits an augmented affinity for some M2 mutants compared to the wildtype M2. Molecular dynamics simulations unveil interactions between amino acids of the helix and the catalytically significant DHHC and TTXE motifs of DHHC20. Our findings suggest that the binding of M2 to DHHC20, while not highly specific, is mediated by requisite contacts, possibly instigating the transfer of fatty acids. A comprehensive comprehension of protein palmitoylation mechanisms is imperative for the development of DHHC-specific inhibitors, holding promise for the treatment of diverse human diseases.

## Introduction

Protein S-acylation (palmitoylation) is the post-translational attachment of fatty acids to the thiol group of cysteine residues via a labile thioester linkage. It is found in intrinsically hydrophilic proteins, where it causes binding to the cytosolic part of various cellular membranes and in transmembrane proteins at membrane-near cysteine residues, where it is often required for targeting to membrane domains. For many cellular proteins, S-acylation is reversed by acyl protein thioesterases. S-acylation regulates a variety of important cellular processes at the membrane-cytosol interface; their malfunctioning has been implicated in a variety of diseases^1,2^.

S-acylation is catalysed by members of the DHHC-family of protein acyl transferases. The 23 human DHHC proteins are polytopic membrane proteins containing an Asp-His-His-Cys (DHHC) motif as the catalytic centre in one of their cytoplasmic domains, which is embedded within a cysteine-rich domain (CRD). Besides this cysteine-rich domain little sequence conservation occurs between DHHC proteins. Most DHHCs are abundant in many tissues, where they mainly localize to Golgi membranes, while a smaller number remain in the endoplasmic reticulum (ER), targeted to the plasma membrane or to endosomes^3,4^. Most cellular proteins can be palmitoylated by several, but not each, of the various DHHC proteins, thereby indicating that the enzymes show distinct, partially overlapping substrate specificities^1,5^.

Crystal structures of DHHC 15 and DHHC 20 reveal that four transmembrane helices form a tent-like structure with the DHHC-motif located at the membrane-cytosol interface^6^. The conserved cysteine-rich region forms six β-sheets that coordinate two zinc-ions via histidine and cysteine residues and contains positively charged amino acids that bind the negatively charged phosphates of acyl-CoA^7^. DHHC proteins exhibit a two-step reaction mechanism, in which a fatty acid is first transferred from the lipid donor acyl-CoA to the cysteine of the DHHC motif (auto-acylation) and subsequently to the substrate protein^8,9^. The acylated enzyme intermediate contains the fatty acid inserted into a hydrophobic cavity formed by all four transmembrane regions. The narrow end of this tunnel contains either small or large amino acids that determine the acyl binding specificity, i.e. palmitate (C16) versus stearate (C18)^6^.

**Figure 1:**
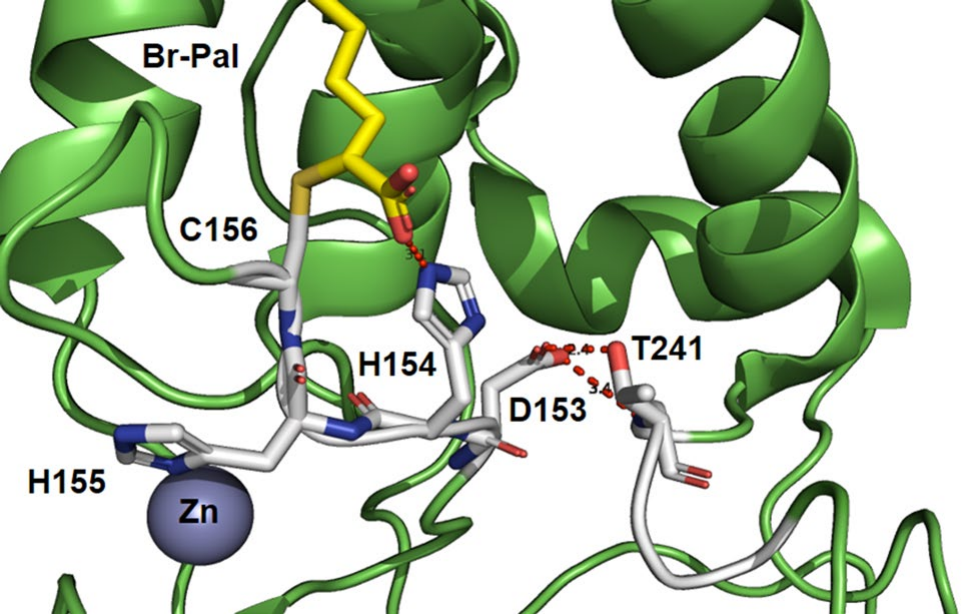
Catalytic centre of the autoacylated form of DHHC20. The amino acids of the DHHC motif and T241 of the TTXE motif are shown as sticks. Incubation with 2-Br-palmitate resulted in alkylation of cysteine 156 through attachment at the α position of palmitic acid. *Zn* zinc ion. Created with PyMol from pdb-file 6BML.

The catalytic mechanism of palmitoylation is a nucleophilic substitution reaction. In the case of autoacylation of DHHC enzymes, the -SH group of the cysteine in the _153_Asp-His-His-Cys_156_ (DHHC) motif must be deprotonated. The resulting thiolate then acts as a nucleophile and attacks the carbonyl carbon of Pal-CoA^10,11^. To initiate the palmitoylation reaction Asp153 needs to polarise the neighbouring His154, which then acts as a base and deprotonates Cys156. The other His155 of the DHHC motive coordinates one of the two Zinc ions, which are important for the structural integrity of DHHC proteins^12^. Downstream of TM4 but spatially close to the DHHC-motif is a conserved _240_Thr-Thr-X-Glu_243_ (TTXE) motif, which is also important for catalysis^4^. The structure of autoacylated DHHC20 shows that the side chain of Thr241 interacts with the carboxylate of Asp153, hindering its function for the deprotonation of His154 (Fig. 1). To transfer the fatty acid from DHHC to a substrate protein these hydrophilic interactions need to be remodelled.

The molecular mechanism by which substrates are recognized and enzyme catalysis is initiated is poorly understood. Several DHHCs contain interaction domains, such as ankyrin repeats, PDZ-binding motifs and SH2 domains with which they recruit (mainly intrinsically hydrophilic) substrates. Only one structure of such a complex is known, namely for an N-terminal ankyrin domain of DHHC17 bound to a peptide from the substrate SNAP 25^13^. How transmembrane proteins are recognized by DHHC enzymes is essentially unknown and there is no consensus sequence that directs palmitoylation^10,14,15^. However, there is increasing evidence that in various proteins an amphiphilic helix, often located near a transmembrane region, serves as a recognition feature for DHHC20 and other DHHCs, but no molecular structural information is available for any of these helices^16–20^.

Protein palmitoylation was first described for viral spike proteins and they have been important in elucidating basic biochemical features of S-acylation^21–23^. Here we used the short ion channel M2 of Influenza A virus to determine molecular determinants of palmitoylation. We have recently shown that DHHC 2, 8 15 and 20 are required for acylation of M2 and a similar set of DHHC proteins is involved in acylation of the spike glycoprotein hemagglutinin (HA) of the same virus^24^. M2 consists of a short N-terminal domain exposed extracellularly, a transmembrane segment of 19 amino acids and a 54-residue cytoplasmic tail. The M2 protein is expressed abundantly at the cell surface but present in only a few copies in virus particles^25,26^. During virus entry the proton channel activity of M2 acidifies the interior of virus particles which is required for uncoating of the viral genome^27^. During virus budding a membrane-near amphiphilic helix inserts into the cytosolic part of the plasma membrane, which causes membrane curvature required for virus scission^28^. The amphiphilic helix is palmitoylated at one cysteine at its beginning, but removal of the palmitoylation site only subtly affects virus replication^29–31^. The formation of the helix requires binding to lipid membranes, but not fatty acid attachment^32,33^. M2 is of interest since an NMR structure of the transmembrane region and amphiphilic helix is available^33^. This allows not only for targeted mutations but also to develop a model of the DHHC20/M2 complex. Deciphering the mechanism of protein palmitoylation and identifying the contact structures involved is important to develop DHHC-specific inhibitors that interfere with replication of many viruses and target a variety of other human diseases^34,35^.

## Results

### The amphiphilic helix of M2 contains intrinsic signals for palmitoylation

**Figure 2:**
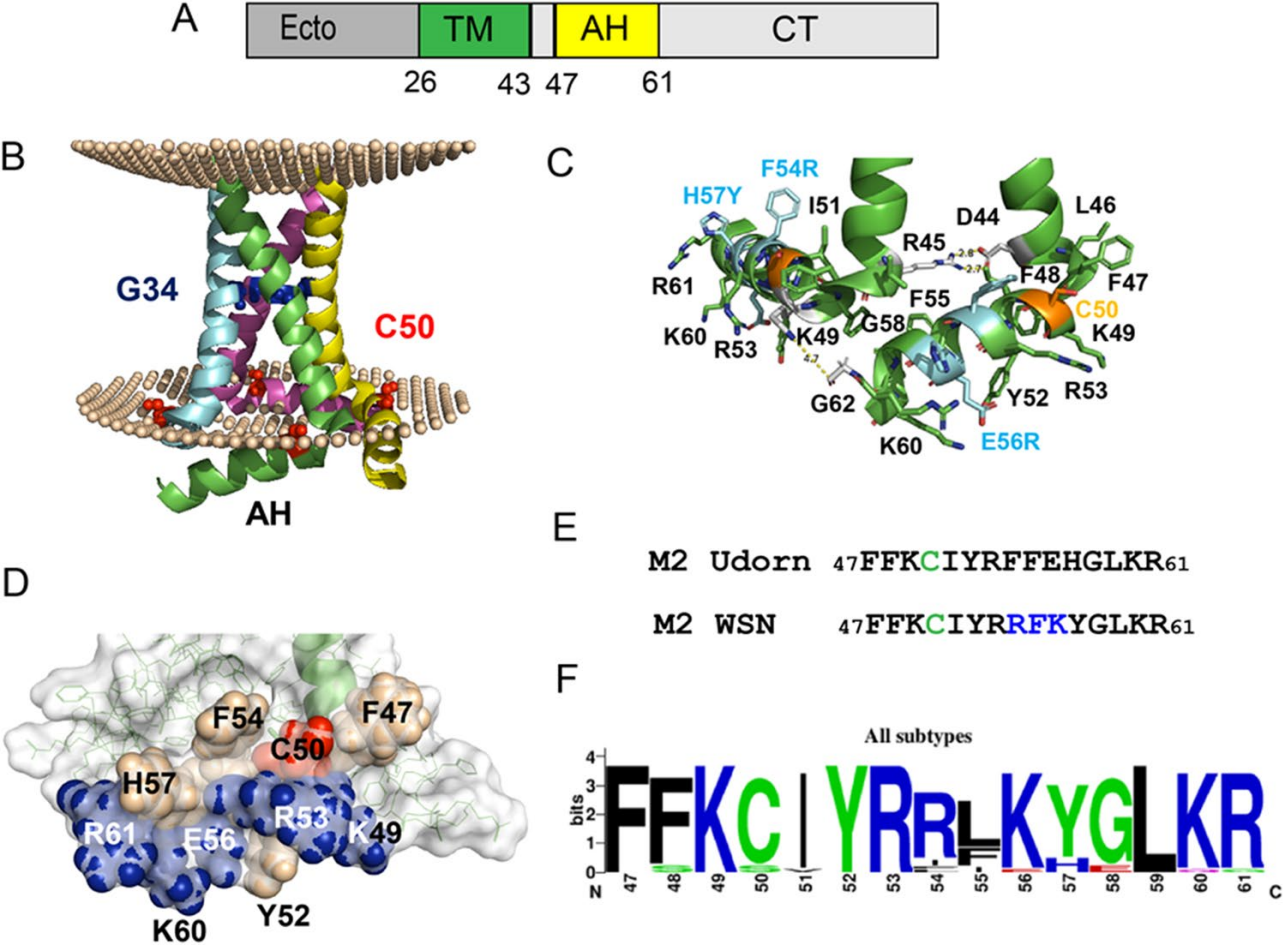
Primary and 3D structure of M2 of Influenza A virus. (**A**) Scheme of M2: Unglycosylated ectodomain, TM: transmembrane region (aa 26–43), AH: amphiphilic helix (47–61) cytoplasmic tail. (**B**) NMR structure of M2 embedded in a virtual lipid bilayer for visualization of the location of the TM and AH. G34: glycine in the middle of the TM slightly bends the helix. C50: acylation site at the beginning of the amphiphilic helix (AH). Note that the M2 used to determine the structure contains a serine instead of a cysteine at position 50. The figure was created from pdb file 2L0J with the PPM 3.0 web-server https://opm.phar.umich.edu/ppm_server3, which calculates the position of a membrane protein within a lipid bilayer. (**C**) Location of amino acid side chains in the structure of the amphiphilic helix. The three residues highlighted in cyan differ between the M2 variants used for NMR and for our experiments. Hydrophilic interactions between D44 and R45 and K49 and G62 connect two monomers. (**D**) Surface representation of the amphiphilic helix. Charged amino acids in blue, aromatic in wheat and acylated Cys50 in red. (**E**) Amino acid sequence of the AH in M2 used for structural analysis (upper row) and in this study (lower row). (**F**) Web-Logo of all M2 sequences shows conserved and variable residues in the amphiphilic helix.

A M2 monomer (97 amino acids) is composed of a short, unglycosylated ectodomain, one transmembrane region (TM, aa 26–43) and a cytoplasmic tail that contains an amphiphilic helix (AH, 47–61) and an intrinsically disordered region (62–80). M2 is a homo-tetramer, which is stabilized by formation of intersubunit disulphide bonds between Cys17 and Cys19 in the ectodomain (Fig. 2A). Figure 2B shows the NMR structure of the TM and AH integrated into a virtual lipid bilayer^33^. The four helical transmembrane domains run through the membrane at an angle and are slightly bent in the middle due to the presence of Gly34, and connected by a short 90° turn to the amphiphilic helix. The helix runs almost parallel to the membrane and contains the acylated Cys50 at the beginning of the hydrophobic face, so the fatty acid can insert into the lipid bilayer. The C-terminal parts of two TMs are connected by an ionic bond between Asp44 and Arg45 and two AHs by a hydrogen bond between Lys49 and Gly62 (Fig. 2C). The AH contains many amino acids with distinctive side chains, such as Phe, Tyr, His, Arg, Lys, which are localized on its surface and could bind to a DHHC enzyme (Fig. 2C and D). Note, however that the sequence of the AH is not conserved through all Influenza strains. The M2 protein we used contains two non-conservative (Phe54Arg, Glu56Arg) and one conservative amino acid exchange (His57Tyr, stained in cyan Fig. 1B) relative to the M2 for which the NMR structure was determined (Fig. 2E). It thus corresponds better to the consensus sequence calculated for all M2 proteins (Fig. 2F).

**Figure 3:**
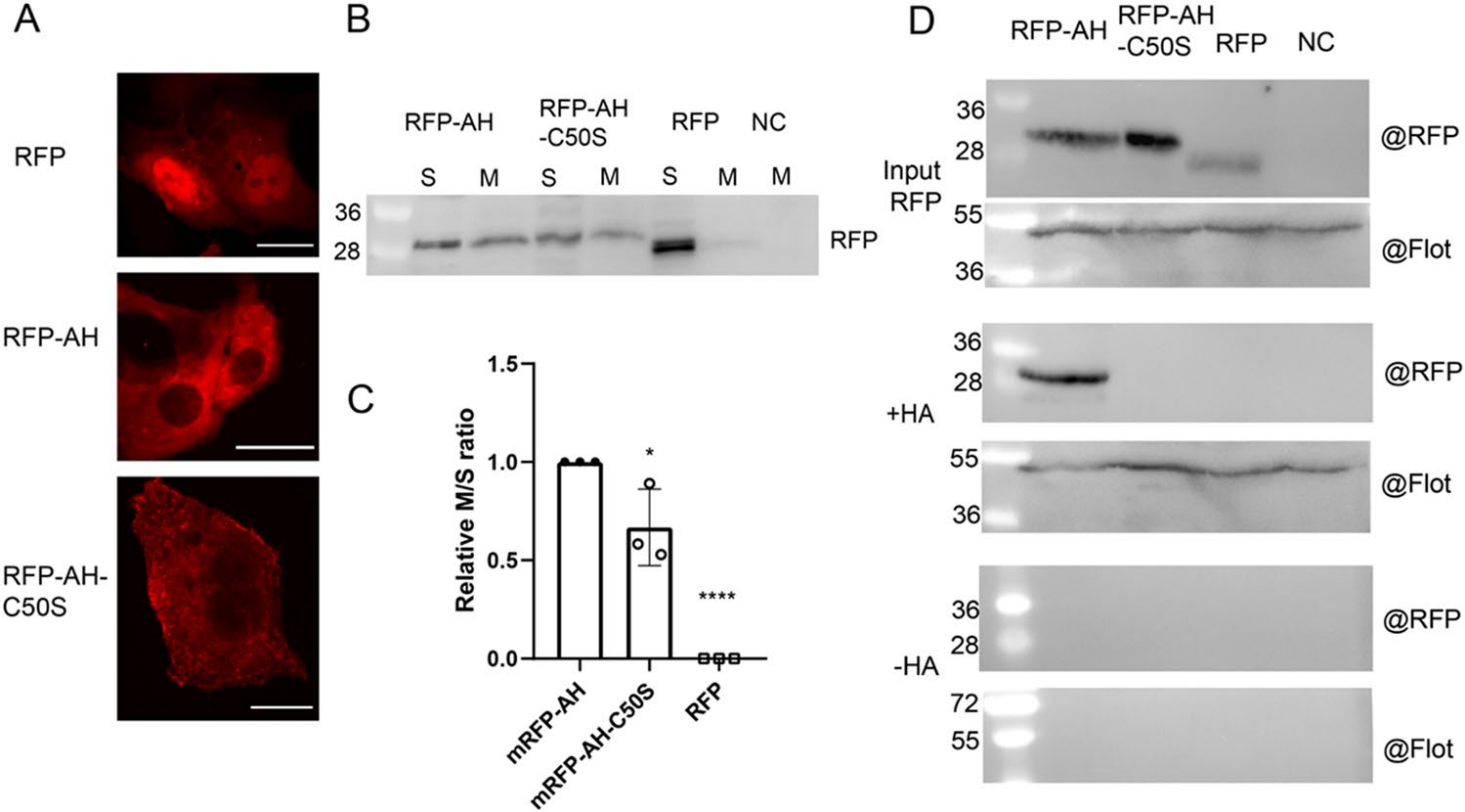
Membrane localization and acylation of the amphiphilic helix of M2 fused to RFP. (**A**) Confocal microscopy: RFP, RFH-AH, RFP-AH-C50S were expressed in BHK21 cells, which were analysed by confocal microscopy. The scale bar is 20 µm. (**B**) Membrane separation experiment: RFP, RFH-AH, RFP-AH-C50S were expressed in 293 T cells. Cells were lysed and separated into soluble (S) and membranous (M) fractions, which were subjected to blotting with anti-RFP antibodies. 10% of the cytosol preparation and 20% of the membranes were analysed in the blot. (**C**) Quantification of this and two other independent experiments. The ratio of the density of the M and S bands was calculated, normalized to RFP-AH (= 1). The mean ± SD and the results from the three independent experiments are shown. One-way ANOVA followed by multiple comparison Dunnett test was applied for statistical analysis. *P < 0.05 P = 0.0195, ****P < 0.0001 versus RFP-AH. (**D**) S-acylation: RFP, RFH-AH, RFP-AH-C50S were expressed in 293 T cells which were lysed 24 h after transfection. To test for protein expression, 10% of the lysate was removed (input). The remainder was divided into two aliquots, one not treated (− HA) and one treated with hydroxylamine (+ HA) to cleave cysteine-bound fatty acids before pulling down proteins with a free SH group. Samples were subjected to Western blotting with antibodies against RFP and, subsequently against flotillin-2, an endogenous acylated protein.

To investigate whether the helix of M2 becomes acylated in the absence of the TM, we fused its sequence, either corresponding to the wt helix or a Cys50Ser mutant, to the C-terminus of the red fluorescent protein (RFP). Confocal microscopy of transfected BHK21 cells revealed, that RFP, which has no intrinsic membrane targeting features, localizes to the cytosol and to the nucleus. In contrast, RFP-AH and RFP-AH-C50S are excluded from the nucleus and redistributed to intracellular compartments, especially to a perinuclear region, which is partially stained by antibodies against GM130, a component of the cis-Golgi, an intracellular site where DHHC20 (in addition to the ER) is located^36^ (Fig. 3A).

We then performed a membrane fractionation assay to analyse the distribution of the proteins between cytosol and membranes. As expected, RFP is present only in the cytosolic fraction, whereas RFP-AH and also RFP-AH-C50S are also membrane-bound,  ~30% of total RFP-AH is in the membrane fraction (Fig. 3B). Thus, the amphiphilic helix of M2 has the capacity to interact with cellular membranes, even in the absence of the acylation site. However, quantification of three independent experiments revealed that membrane binding of RFP-AH-C50S is 35% reduced relative to RFP-AH (Fig. 3C).

To test whether RFP-AH becomes acylated, we used the Acyl-RAC (resin-assisted capture) assay, which exploits thiol-reactive resins to capture SH-groups in proteins. Twenty-four hours after transfection, cells were lysed and 10% of the total extract (TE) was removed from the lysate to determine the expression levels. Disulphide bonds in proteins present in the remaining part were reduced and newly exposed -SH groups were blocked. The sample was then equally split: one aliquot was treated with hydroxylamine (+ HA) to cleave thioester bonds, and the other aliquot was treated as control with Tris–HCl buffer (–HA). After pull-down of proteins with the thiol-reactive resin, samples were subjected to western blotting using antibodies against the RFP-tag. To exclude that proteins were lost during sample preparation, we used antibodies against the cellular palmitoylated protein flotillin 2 as an internal control. The results clearly show that RFP-AH is acylated, since it is precipitated by the thiol-reactive beads, which is not the case for RFP and RFP-AH-C50S (Fig. 3D).

### Mutations in the helix reduce but do not abolish palmitoylation of M2

**Figure 4:**
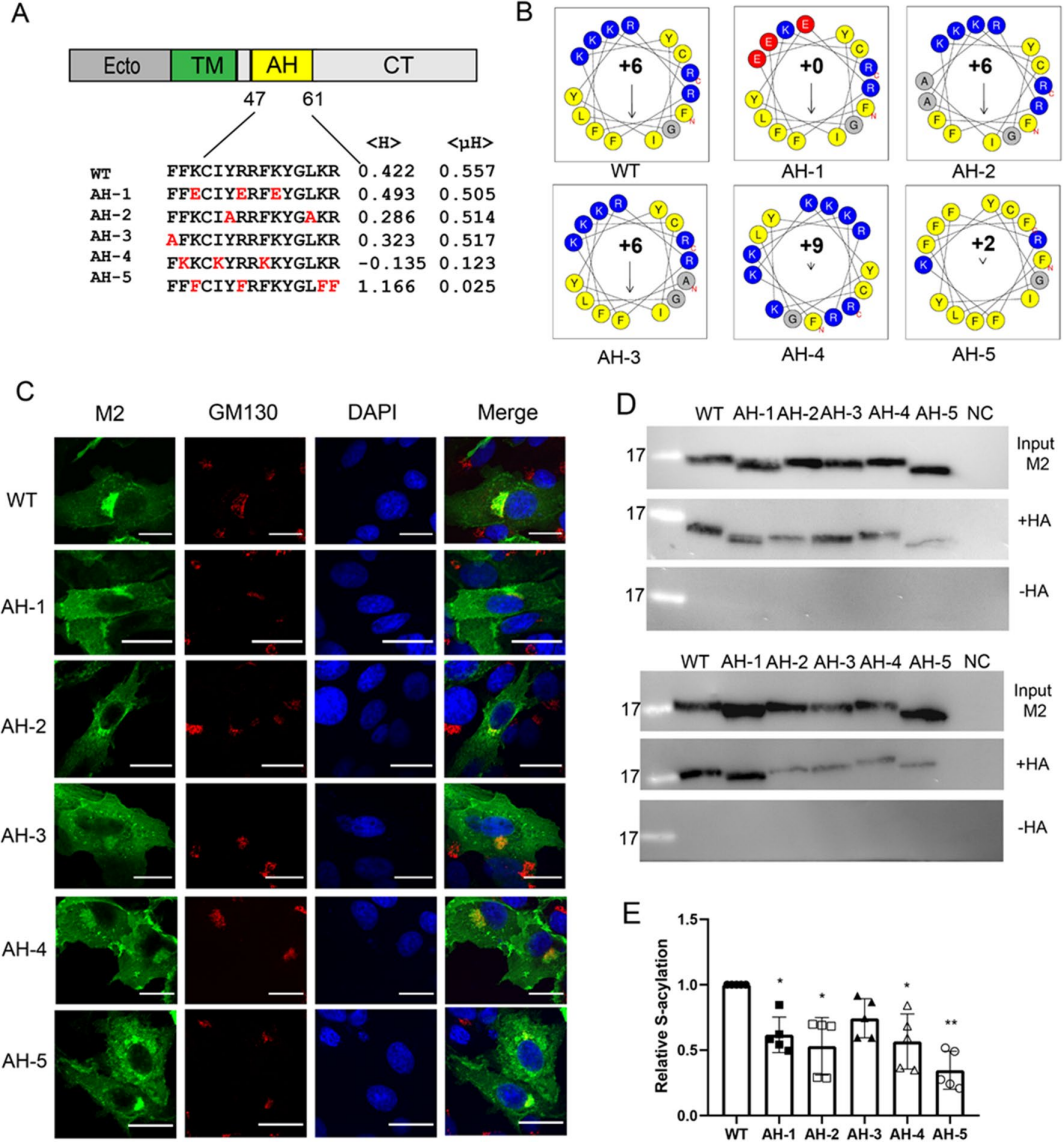
Palmitoylation and intracellular localization of M2 with mutations in the amphiphilic helix that affect its biophysical properties. (**A**) Scheme of M2 and the sequence of the amphiphilic helix with the mutations inserted highlighted in red. The hydrophobic moment < μH > and overall hydrophobicity < H > of the helix were calculated with Heliquest (https://heliquest.ipmc.cnrs.fr/). (**B**) Helical wheel plots of the amphiphilic region of wild type M2 and the M2-AH mutants. Positive amino acids are in blue, negative amino acids in red, and hydrophobic amino acids in yellow. The arrow points to the hydrophobic face. The length of the arrow corresponds to the hydrophobic moment < μH > . The numbers indicate the net charge of the helix. (**C**) Confocal microscopy of mutants in transfected BHK21 cells. Cells were permeabilized and stained with antibodies against M2 (green), the cis-Golgi marker GM130 (red) and with DAPI (blue) to highlight the nucleus. The scale bar is 20 µm. (**D**) S-acylation: M2 wt and the mutants were expressed in 293 T cells. For the input samples different volume of the lysate was removed to adjust for the reduced expression level of some mutants. The remainder was divided into two aliquots that were adjusted to the same extent. One aliquot was not treated (− HA) and one treated with hydroxylamine (+ HA) to cleave cysteine-bound fatty acids before pulling down proteins with a free SH group. Samples were subjected to Western blotting with antibodies against M2. The results of two independent experiments are shown. (**E**) Quantification of these two and two other Acyl-RAC assays. The density of the bands with hydroxylamine (+ HA) bands was divided by the density of the input bands and normalized to M2 wild type (= 1)). The mean ± SD and the results from five independent experiments are shown. One-way ANOVA followed by multiple comparison Dunnett test was applied for statistical analysis. *P < 0.05, **P < 0.01 versus wild type.

We then investigated which features of the helix are essential for acylation of M2. We used the authentic M2 protein, which is targeted by its transmembrane region to the ER and then transported through the exocytic pathway to the plasma membrane such that all molecules have access to DHHC enzymes^25,37^. We exchanged amino acids exposed at the molecule’s surface, that alter the charge of the helix and/or its biophysical properties, such as hydrophobicity and hydrophobic moment. In M2 AH-1 the positively charged Lys49, Arg53 and Arg56 exposed at the frontside of the AH were exchanged by negatively charged Glu (Fig. 4A, see Supplementary Fig. 1 for localization of mutated amino acids in a surface representation of M2). These substitutions do not greatly alter the hydrophobicity and the hydrophobic moment of the helix as calculated by the tool heliquest, but they eliminate its positive net charge (Fig. 4B). In M2-AH-2, two long hydrophobic residues (Tyr52, Leu59) located at the back bottom of the AH were replaced by less hydrophobic alanine residues. In M2-AH-3, Phe47, which forms a distinct protrusion at the beginning of the helix near the acylated cysteine, has been replaced by alanine. The helices of M2-AH-2 and M2-AH-3 are less hydrophobic, but still exhibit an amphiphilic character. M2-AH-4 contains three positively charged lysine instead of three hydrophobic residues (Phe48, Ile51, Phe55), located at the back bottom of the helix. In AH-5, four positively charged residues (Lys49, Arg53, Lys60, Arg61), which are exposed at the frontside of the AH, were replaced by Phe. The mutations in M2-AH-4 and M2-AH-5 essentially destroyed the amphiphilic character of the helix. AH-4 is hydrophilic and AH-5 is more hydrophobic compared to the wt-helix.

The resulting mutants were expressed in 293 T cells, which were analysed by western blotting with M2 antibodies. The signals of M2-AH-1, M2-AH-3 and M2-AH-5 are substantially reduced compared to those of M2 wt and the other mutants (Supplementary Fig. 2). We were concerned that the mutations would lead to misfolding and subsequent degradation of M2 by the cell’s quality control system, which would manifest as blockage of transport out of the ER^38^. Confocal microscopy revealed for M2 wt and all mutants reticular staining in the cytosol, likely representing the ER, transport to the plasma membrane and especially bright perinuclear staining, which overlaps with the cis-Golgi marker GM 130 (Fig. 4C). Thus, the M2 mutant proteins expressed are localized to the same compartments as M2 wt. Calculation of the Pearson’s correlation coefficient revealed that four M2 mutants co-localize to the same extent as M2 wt with the marker GM130. Only transport of M2-AH-4 to the cis-Golgi was slightly reduced (Supplementary Fig. 9). Therefore, the mutations are unlikely to cause misfolding of M2 and a defect in its transport to the intracellular acylation site.

However, to compare the acylation of the M2-AH mutants, we had to adjust the amount of cell lysate used as input and accordingly the amount incubated with the thiol-reactive beads to account for the different expression levels. This prevented us from using flotillin as internal acylation control. Instead, we performed each Acyl-Rac assay four times to compensate for random loss of protein during sample preparation. Two of the results are shown in Fig. 4D to demonstrate the variability between experiments. The quantification of the five experiments (ratio of hydroxylamine-treated to input M2 normalized to M2 wt) is shown in Fig. 4E. Each mutation reduced acylation of M2 in each experiment. The reduction was greatest in M2-AH5 (reduced to 30% relative to M2 wt), followed by M2-AH-2 (50%), M2-AH-4 (54%) M2-AH-1 (62%) and least in AH-3 (75%). Although the differences between the experiments are relatively large, the mean values for most mutants (with the exception of M2 AH-3) were statistically significantly different from those for M2 wt.

### Truncation and destruction of the helix reduces, but does not abolish palmitoylation of M2

**Figure 5:**
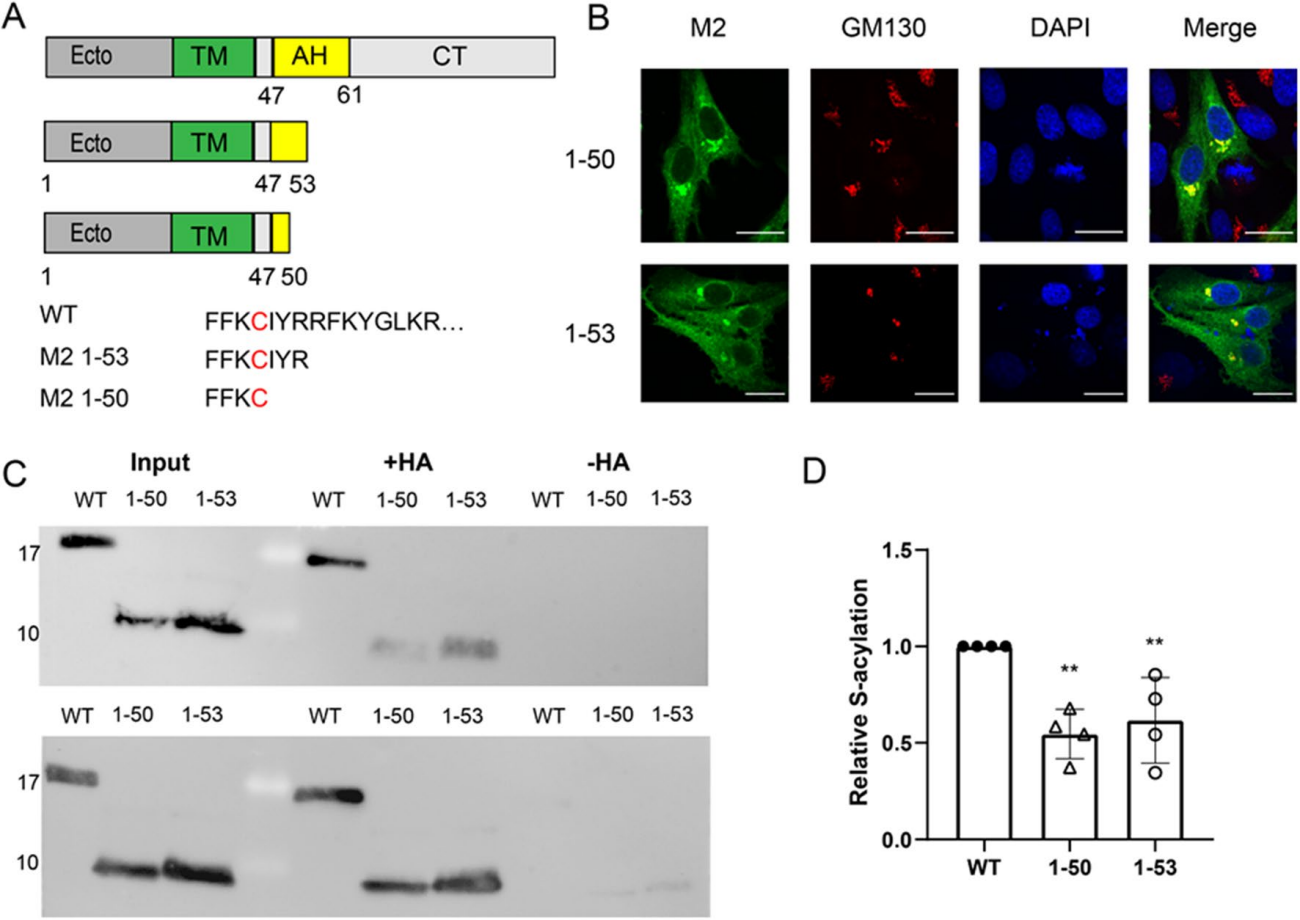
Palmitoylation and intracellular localisation of M2 mutants with truncated amphiphilic helix. (**A**) Scheme and sequence of M2, wild type and truncated mutants. (**B**) Confocal microscopy of mutants in transfected BHK21 cells. Cells were permeabilized and stained with antibodies against M2 (green), the cis-Golgi marker GM130 (red) and with DAPI (blue) to highlight the nucleus. The scale bar is 20 µm. (**C**) S-acylation: M2 wt and the mutants were expressed in 293 T cells. For the input samples, different volume of the lysate was removed to adjust for the reduced expression level of some mutants. The remainder was divided into two aliquots that were adjusted to the same extent. One aliquot was not treated (− HA) and one treated with hydroxylamine (+ HA) to cleave cysteine-bound fatty acids before pulling down proteins with a free SH group. Samples were subjected to Western blotting with antibodies against M2. The results of two independent experiments are shown. (**D**) Quantification of these two and two other independent experiments. The density of the bands with hydroxylamine (+ HA) bands was divided by the density of the input bands and normalized to wild type (= 1)). The mean ± SD and the results from four independent experiments are shown. One-way ANOVA followed by multiple comparison Dunnett test was applied for statistical analysis. **P < 0.01 versus wild type.

We investigated next whether a helical structure downstream of Cys50 is required for the basal acylation of M2. We created two mutants where the cytoplasmic tail of M2 including most parts of the helix were deleted. Mutant M2 1–50 retains the first four helical residues including Cys50, M2 1–53 contains three additional residues downstream of the acylation site (Fig. 5A). M2 1–53 and especially M2 1–50 are expressed at lower levels than M2 wt (Supplementary Fig. 2). However, confocal microscopy revealed for both mutants the staining pattern typical for M2, including strong perinuclear staining. M2 1–50 even showed slightly enhanced co-localization with the cis-Golgi marker compared to M2 wt and M2 1–53 (Fig. 5B, quantification in Supplementary Fig. 9). The Acyl-RAC assay showed that the acylation of the mutants is greatly reduced but still clearly detectable (Fig. 5C). Quantification of four experiments showed a statistically significant reduction of about 45% for M2 1–50 and 40% for M2 1–53, but with relatively large variation between experiments (Fig. 5D).

**Figure 6:**
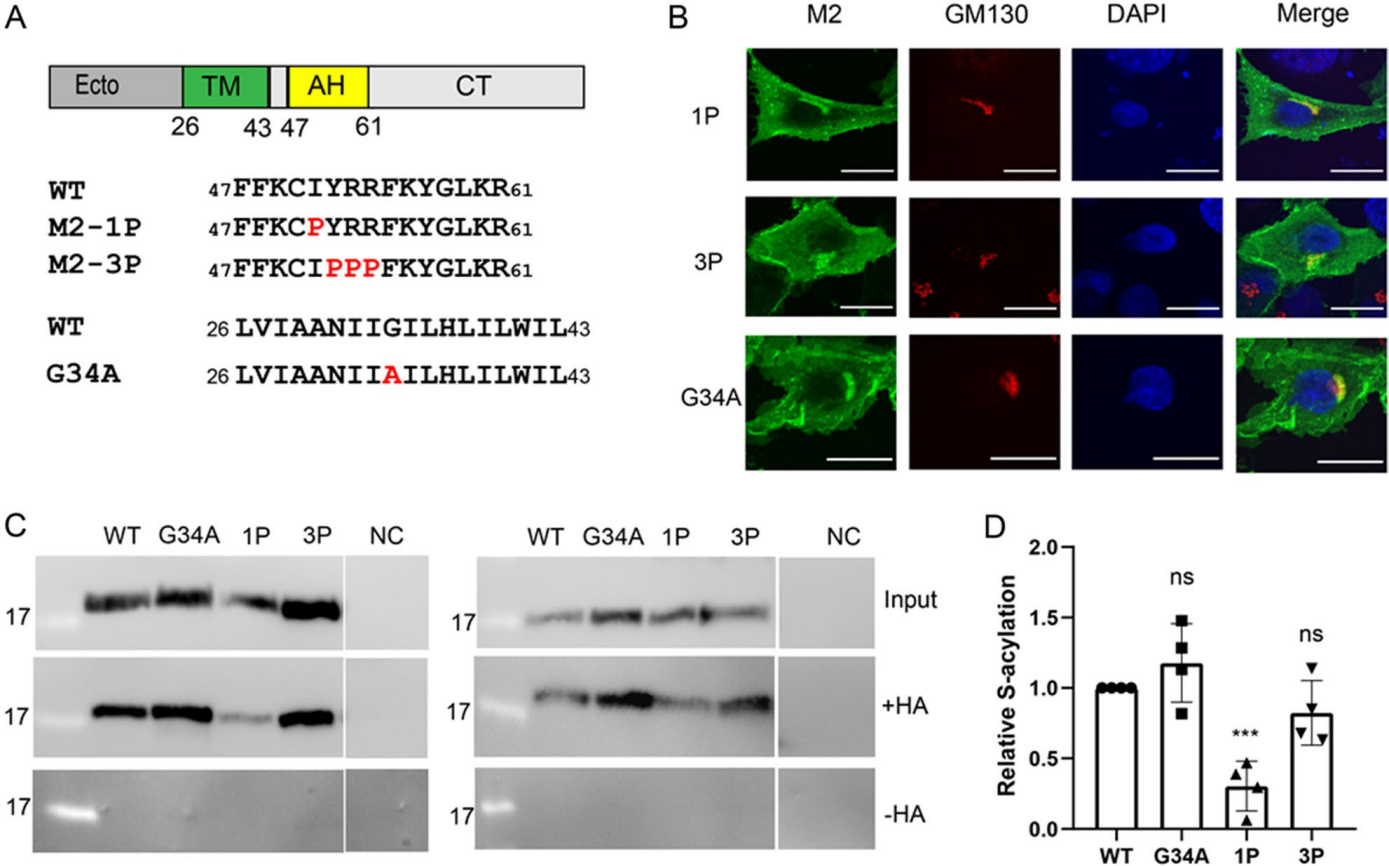
Palmitoylation and intracellular localization of M2 mutants with disrupted amphiphilic helix and replacement of a glycine in the transmembrane region. (**A**) Scheme and sequence of M2, wild type and mutants with one or three Pro inserted into the helix and with the Gly34Ala exchange in the TM. (**B**) Confocal microscopy of mutants in transfected BHK 21 cells. Cells were permeabilized and stained with antibodies against the cis-Golgi marker GM130 (red) and with DAPI (blue) to highlight the nucleus. The scale bar is 20 µm. (**C**) S-acylation: M2 wt and the mutants were expressed in 293 T cells. For the input samples, different volume of the lysate was removed to adjust for the reduced expression level of some mutants. The remainder was divided into two aliquots that were adjusted to the same extent. One aliquot was not treated (− HA) and one treated with hydroxylamine (+ HA) to cleave cysteine-bound fatty acids before pulling down proteins with a free SH group. Samples were subjected to Western blotting with antibodies against M2. The results of two independent experiments are shown. (**D**) Quantification of 5C and two other independent experiments. The density of the bands with hydroxylamine (+ HA) bands was divided by density of the input bands and normalized to wild type (= 1)). The mean ± SD and the results from four independent experiments are shown. One-way ANOVA followed by multiple comparison Dunnett test was applied for statistical analysis. *ns* not significant, ***P < 0.001 versus wild type.

Two further mutants were produced where we inserted helix-destroying proline residues. In the M2-1P mutant, Ile 51 following the acylated cysteine was replaced by a proline. The M2-3P mutant contains three prolines instead of Tyr52, Arg53 and Arg54 (Fig. 6A). Only mutant M2-1P was expressed at lower level (Supplementary Fig. 2), but it co-localizes with the cis-Golgi marker to the same extent as M2-3P and M2-wt (Fig. 6B, quantification in Supplementary Fig. 9). The Acyl-RAC assay showed that the acylation of the mutant M2-1P is greatly reduced to 30% relative to M2 wt. An effect of the insertion of three Pro is less obvious. The mean value of the degree of acylation from four experiments is 82%, but the results are not statistically significant different from M2 wt (Fig. 6C,D).

These results confirm that an amphiphilic helix is essential for efficient acylation of M2, but that a basal level of acylation is maintained even in the absence of a helical structure downstream of the acylation site. This suggests that amino acids in the transmembrane region of M2 might also affect the acylation reaction.

### Replacement of kink-inducing glycine in the transmembrane helix increases palmitoylation of M2

The transmembrane region of M2 (sequence LVIAANII**G**IL**H**LIL**W**IL) contains many of the hydrophobic amino acids commonly found in such a region, which are unlikely to be a specific recognition feature for a DHHC enzyme. Two particular and conserved residues are His37 and Trp41 but their side chains are pointing into the pore formed by tetrameric transmembrane region, and they are known to be essential for proton transport. One peculiar feature of the transmembrane region is a highly conserved glycine in its middle that causes a slight kink in the helix^33^. We changed the helix-disfavouring glycine to an alanine, a common amino acid in helical transmembrane regions. The resulting mutant M2-G34A is expressed at a higher level as M2 wt (Supplementary Fig. 2) and shows the same intracellular staining pattern in confocal microscopy (Fig. 6B, quantification in Supplementary Fig. 9). Quantification of four Acyl-Rac assays indicates that the acylation level of M2-G34A might be slightly higher than that of M2-wt (118%), but the difference was not statistically significant (Fig. 6C,D).

**Figure 7:**
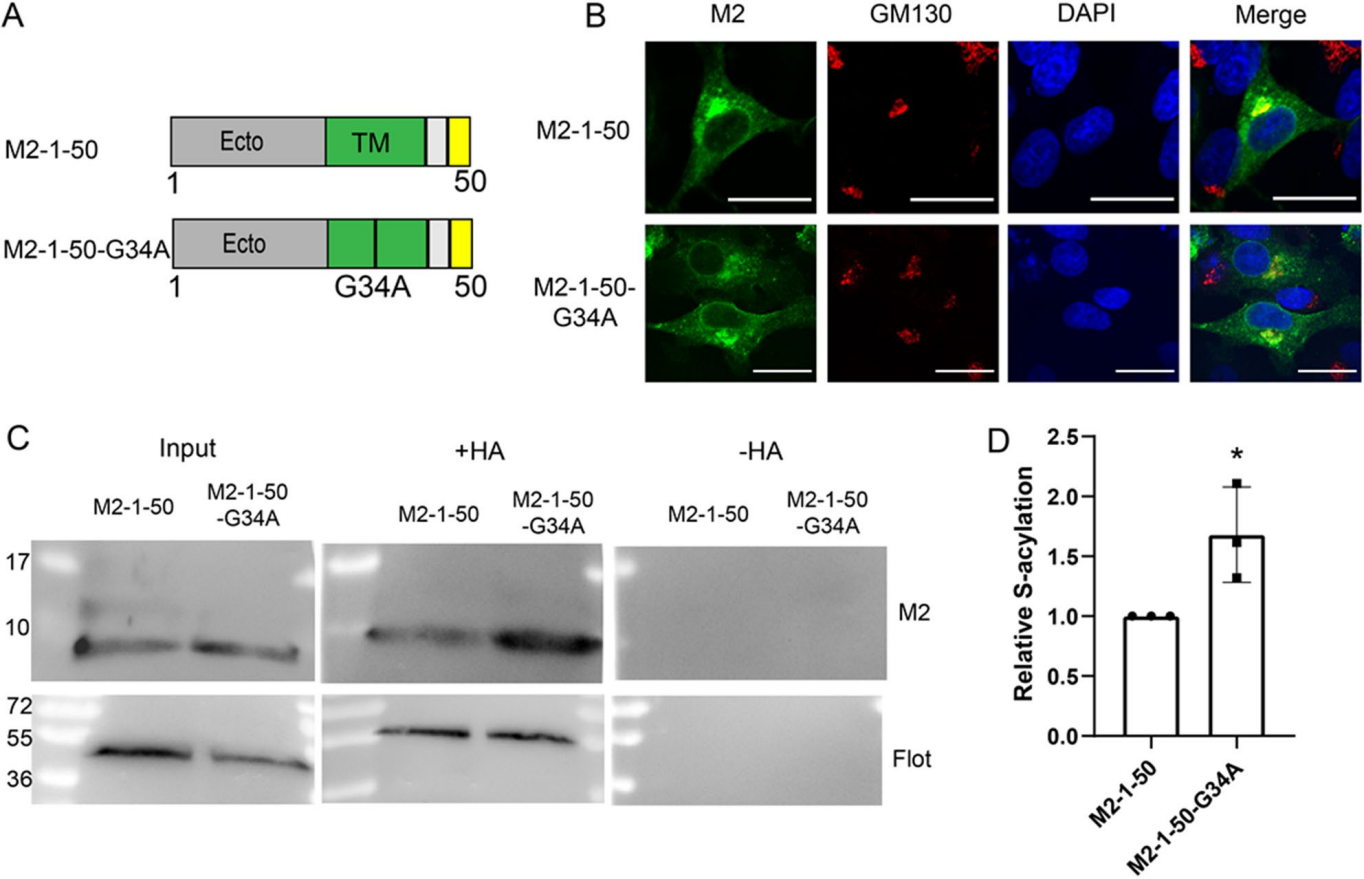
Palmitoylation and intracellular localization of M2 mutants with truncated amphiphilic helix and replacement of a glycine in the transmembrane region. (**A**) Scheme and sequence of M2 1–50, wild type and mutants with the Gly34Ala exchange in the TM. (**B**) Confocal microscopy of mutants in transfected BHK cells. Cells were permeabilized and stained with antibodies against the cis-Golgi marker GM130 (red) and with DAPI (blue) to highlight the nucleus. The scale bar is 20 µm. (**C**) S-acylation: M2 1–50 and M2 1-50-G34A were expressed in 293 T cells. For the input samples, same volume of the lysate was removed to detect expression level. The remainder was divided into two aliquots. One aliquot was not treated (− HA) and one treated with hydroxylamine (+ HA) to cleave cysteine-bound fatty acids before pulling down proteins with a free SH group. Samples were subjected to Western blotting with antibodies against M2 and, subsequently against flotillin-2, an endogenous acylated protein. (**D**) Quantification of 6C and two other independent experiments. The density of the samples with hydroxylamine (+ HA) bands was divided by density of the input and normalized to wild type (= 1)). The mean ± SD and the results from three independent experiments are shown. Unpaired t-test was applied for statistical analysis. *P < 0.05, versus M2-1-50.

We suspected that the relevance of the transmembrane region on acylation would become more pronounced if we minimise the influence of the helix. Thus, we introduced the G34A mutation into the truncated mutant M2 1–50 (Fig. 7A). Both proteins show a similar intracellular staining pattern in confocal micrographs (Fig. 7B). The Acyl-RAC assay shows that both proteins are expressed at the same level (input in Fig. 7c), but the M2-1-50-G34A band in the + HA samples is more intense than the M2-1-50 band. Quantification of three different experiments revealed a 50% increase in acylation of M2-1-50-G34A (Fig. 7D).

### Mutations in the helix of M2 do not prevent binding to DHHC20

**Figure 8:**
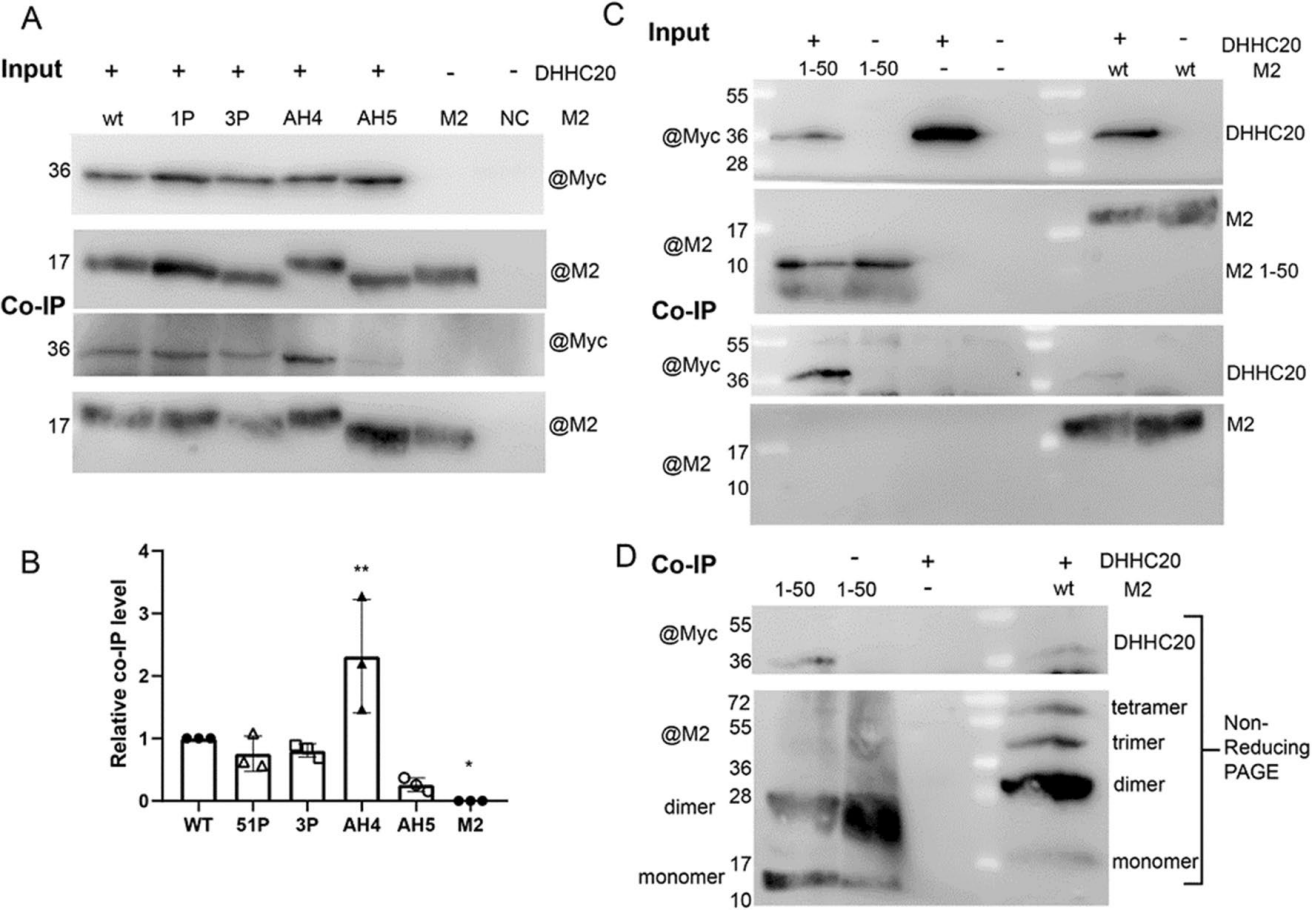
Co-precipitation of DHHC20 with M2 wt and M2 mutants with reduced acylation. (**A**) Input: DHHC20-myc and M2, either wild type and the indicated mutants were expressed in 293 T cells. Cells were lysed with non-denaturating detergent, different volumes of the lysate were removed to adjust for the reduced expression level of especially M2 AH-5 and samples were subjected to reducing SDS-PAGE and blotting with anti-myc and anti M2 antibodies. Co-IP: The remainder of the lysate was divided into two aliquots that were adjusted to the same extent. Samples were then subjected to immunoprecipitation with M2 antibodies and to reducing SDS-PAGE and blotting with anti-myc and anti-M2 antibodies. The Co-IP-blot probed with anti-myc antibodies was exposed 5 times longer than the input blot. (**B**) Quantification of this and two other independent experiments. One-way ANOVA was applied for statistical analysis. *ns* not significant, *P < 0.05, **P < 0.01 versus wild type. (**C**) DHHC20-myc and M2 wild type and M2 1–50 were expressed in 293 T cells. Cells were lysed with non-denaturating detergent, different volumes of the lysate were removed to adjust for the reduced expression level of M2 1–50 and samples were subjected to reducing SDS-PAGE and blotting with anti-myc and anti M2 antibodies. Co-IP: The remainder of the lysate was divided into two aliquots that were adjusted to the same extent. Samples were then subjected to immunoprecipitation with M2 antibodies and to reducing SDS-PAGE and blotting with anti-myc and anti-M2 antibodies. (**D**) Co-IP from C subjected to non-reducing SDS-PAGE.

M2 wt substantially overlaps with DHHC20 in co-transfected BHK21 cells revealing mainly a reticular ER-like and perinuclear staining pattern (Supplementary Fig. 3). Some substrates can be co-immunoprecipitated with their cognate DHHC indicating a long-term, stable interaction during the enzymatic reaction^39^. We asked whether this also applies for the M2-DHHC20 complex and how the most severe helix mutations, M2-1P, M2-3P, M2-AH4 and M2-AH5 affect this interaction. Since the specificity of most commercially available antibodies against DHHCs have not been validated, we used a plasmid encoding human DHHC20 fused at its C-terminus to a myc-tag. DHHC20-myc and M2 were co-expressed in 293 T cells, which were lysed 24 h later. An aliquot of the lysate (5–10%) was removed to monitor the expression level (input). The remainder was subjected to immunoprecipitation with M2 antibodies and the precipitates were blotted with anti-myc and anti-M2 antibodies (Co-IP). Since the expression levels of some M2 mutants, especially M2-AH5, were lower, we adjusted the amount of lysate incubated with the anti-M2 antibodies to provide the same amount of protein. Roughly 1% of total DHHC20-myc was co-precipitated with M2-wt and the M2 mutants also interact with DHHC20-myc (Fig. 8A). The experiments were performed three times and the ratio of co-precipitated DHHC20-myc and M2 were calculated and normalized to the values of M2-wt (Fig. 8B). The results show that the introduction of helix-breaking proline residues does not affect the interaction with DHHC20-myc, but AH-4 and AH-5 have a clear but opposite effect on DHHC20 binding. It is reduced to ~ 20% for M2-AH-5, but increased around twofold for M2-AH-4.

Finally, we analysed the truncated M2 mutant 1–50 for binding to DHHC20-myc. Blotting the M2 antibody precipitate with myc antibodies shows the DHHC20-myc band, even stronger than the DHHC20-myc band obtained after co-expression with M2 wt. Surprisingly, no M2 1–50 was detected, (after longer exposure very little was found), when probing the same membrane with M2 antibodies, although both M2 wt and M2 1–50 are detected at similar levels in the input (Fig. 8C). However, when the M2 antibody precipitate was subjected to non-reducing SDS-PAGE, M2 1–50 can be clearly detected, mainly as a disulphide-linked dimer. M2 wt also appears mainly as a dimer, but also forms some disulphide-linked trimers and tetramers, as already described^40^. The DHHC20-myc band is also present in samples separated by non-reducing SDS-PAGE, and to a slightly higher extent if co-expressed with M2 1–50 (Fig. 8D). It thus appears that M2 1–50 forms unusual large aggregates during immunoprecipitation that fail to penetrate the gel under reducing conditions. Nevertheless, M2 1–50, which basically consists of one transmembrane region binds to DHHC20 inside cells.

The experimental results are summarized in Supplementary Table 1. We conclude that most M2 mutants can form an enzyme–substrate complex, but that the subsequent steps of the enzymatic reaction, fatty acid transfer or, in the case of mutant AH4, perhaps release of the M2 protein from the enzyme, are impaired.

### Molecular modelling of the M2 DHHC 20 interaction

**Figure 9:**
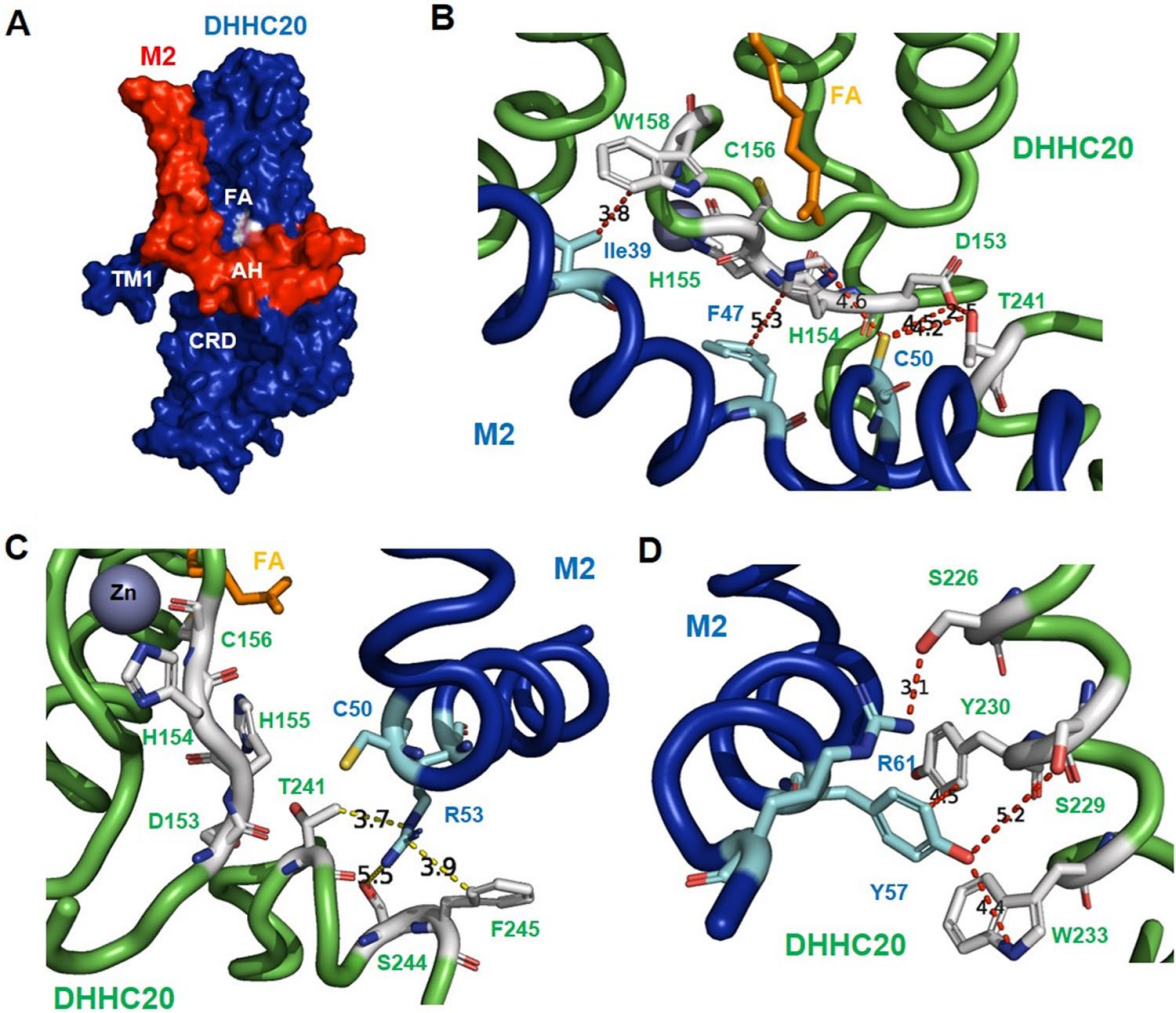
Molecular dynamics simulation of the M2 DHHC20 complex. (**A**) Surface representation: M2 in red, AH: amphiphilic helix. DHHC20 in blue. CRD: cysteine-rich domain in the cytoplasmic domain. TM1: transmembrane region 1. See Supplementary Fig. 4 for a visualization of the interacting amino acids in the entire molecule of DHHC20 and M2. (**B**–**D**) Residues in the amphiphilic helix of M2 (blue) that contact residue in DHHC20 (green) important for catalysis. Residues in M2 are highlighted as cyan sticks and in DHHC as white sticks. The distance is indicated in angstroms. Orange stick: Fatty acid. Zn: Zinc ion. One snapshot of the MD simulation is shown. Note that the helix has a kink downstream of Cys50 which appears to remain during the course of the simulation.

To identify the contact surface between M2 and DHHC20 we performed molecular dynamics simulations. A fully resolved docked structure is currently unknown so we follow the steps outlined in the methods section to generate an initial starting representation. A snapshot of the simulation of the M2 DHHC20 complex is shown as a surface representation in Fig. 9A. The figure shows that the amphiphilic helix of M2 docks to a 25 Å wide cavity in the cytoplasmic domain of DHHC20 close to its transmembrane region. Contact analysis, as described in the methods section, shows that the proteins form 18 interactions involving 13 residues in DHHC20 and 12 residues in M2 (highlighted in both structures in Supplementary Fig. 4 and listed in Supplementary Table 2). These interactions can be broken into interactions residing in the transmembrane and amphiphilic sections of M2. In the transmembrane helix four residues of M2 interact with four residues located on the outside of TM1 of DHHC20. Ile39 of M2 also interacts with Trp158, which is located near the hydrophobic tunnel and is supposed to act as a gate (Fig. 9B)^41^.

The other residues in M2 contacting DHHC20 are located in its amphiphilic helix. Interestingly, the _153_DHHC_156_ motif of DHHC20 has contact to several M2 residues: M2 Cys50 (the acylation site) contacts DHHC residue Asp153 & His154 and M2 Phe47 with DHHC His154 & His155 (Fig. 9B). Likewise, Thr241 in the conserved _240_TTXE_243_ motif is in contact with both Cys50 and Arg53 (Fig. 9C). The C-terminal part of the helix wraps around TM4 of DHHC20 where Tyr57 contacts Ser229, Tyr 230 and Trp 233 (Fig. 9D).

We also performed MD simulations with different M2 mutants to observe structural differences in binding sites, either by deletion of the amphiphilic helix or single amino acid substitutions as described in the methods section. As we have no direct way with classical MD simulations to observe the transfer of the fatty acid from DHHC to M2, the distance between M2 Cys50 and DHHC Cys156 is used as a proxy for binding affinity. If the distance between the residues remains within the bounds of thermal fluctuations in comparison to the wt, we can suspect that transfer could occur on the given timescale.

A plot of this Cys-Cys distance as a function of simulation length is shown in Supplementary Fig. 5A and we observe that for the M2 1–50 (red line) this distance jumps to values to over 15 Å over the course of 100 ns and drifts to 20 Å in the second half of the simulation. Examination of the trajectory shows that the truncated M2 molecule starts to align along the transmembrane domain of DHHC20 (Supplementary Fig. 5B) forming new contacts listed in Supplementary Table 3. The M2 Ile51Pro (orange) and Ile51Ala (green) mutants remain within a similar range (7–10 Å) to the wt. While the distance is conserved observation of the trajectories highlights a decrease in angle between the transmembrane region and the helix which we attribute to the changed geometry of the M2 mutants (Supplementary Fig. 6). Contact analysis shows that the contact of Phe42 in M2 with His 154/155 of the DHHC motif is shifted to preferential contacts with Pro157/Trp158 in Ile51Pro and in Ile51Ala. Likewise, the contact between Arg53 of M2 and Thr241 of the TTXE motive is reduced by ~ 60/30% in the Ile51Pro/Ile51Ala relative to the wt (Supplementary Table 4).

In addition to the Cys-Cys distance analysis, we directly estimate the free energy of binding for a Arg53Ala mutant by construction of a thermodynamic cycle as outlined in the method section. This residue was selected for mutation as Arg53 shows a high degree of contact from the wt trajectory and is in part responsible for the amphiphilic nature of the helix. In addition, the AH-5 experimental mutation showed substantial reduction in expression. However, we observe that strikingly the Arg53Ala mutant has an increased binding affinity to DHHC over wt by 3.0 ± 0.7 kcal/mol. A similar contact analysis at the edges of the switching parameter show that the Arg53Ala mutation has generally stronger contacts overall, in particular for residues Phe47, Cys50, and Arg54 (Supplementary Table 5). Analysis of the radius of gyration of the M2 only trajectory, i.e. no DHHC present, show that the wt prefers to adopt a more extended structure (${R}_{g}=17.89\pm 0.09$Å) but the Arg53Ala mutant more readily adopts the compact structure (${R}_{g}=17.3\pm 0.3$Å) that is more indicative of the M2/DHHC complex (Supplementary Fig. 7).

## Discussion

### The amphiphilic helix of M2 contains all the required information for palmitoylation

The amphiphilic helix of M2 becomes palmitoylated when fused to the C-terminus of the red fluorescent protein indicating that the information for fatty acid attachment is encoded in the helix. The helix also redistributes the reporter protein from its usual nuclear and cytosolic location to cellular membranes. This also occurs, although is reduced by about 30%, when the acylation site is replaced by a serine (Fig. 3). Thus, the helix of M2 has intrinsic propensity to associate with cellular membranes, which is then stabilized by palmitoylation^28,42^. A substantial proportion of RFP-AH (~ 70%) is located in the cytosol, making it unlikely that acylation is stochiometric, since palmitoylated molecules are (almost) always membrane-bound. There are several mutually exclusive explanations for this observation: some of the fatty acids are cleaved by thioesterases, the initial palmitoylation is substoichiometric because the AH does not contain all the information for complete acylation, and/or due to incomplete targeting to membranes not every molecule has access to the membrane-bound DHHC.

### Mutations in the amphiphilic helix reduce, but do not eliminate palmitoylation of M2

To further investigate the signals in the AH, we used the full-length M2 protein, as it is transported to the plasma membrane via the exocytic pathway and thus has complete access to DHHC enzymes^25^. As described previously^43^ some of the mutations reduced the expression level of M2 but this is unlikely to be due to misfolding, since all mutants were transported along the exocytic pathway and co-localized with a cis-Golgi marker to the same extent as M2 wt. Each mutation we introduced into the amphiphilic helix reduced the acylation of M2, but to varying degrees, from 30 to 82% compared to M2 wt (Figs. 4, 5, 6, results are summarized in Supplementary Table 1). The strongest reduction to 30% was achieved by eliminating the hydrophobic moment of the AH, increasing its hydrophobicity (M2 AH-5), and by inserting a proline adjacent to the acylated cysteine (M2-1P). The latter mutant still contains all amino acids identified by molecular dynamics to interact with DHHC20. In M2 AH-4, none of the interacting amino acids were exchanged either, although acylation was determined to be reduced by about 50%. This indicates that the conformation of the helix and its biophysical properties, such as hydrophobicity and hydrophobic moment play a role for efficient acylation. Note, that the inherent variability of the Acyl-RAC assay makes it difficult to assess whether certain mutations have stronger effects than others. The acylation level of two mutants, M2 AH-3 (to 74%) and M2-3P (to 82%), was not statistically significantly different from M2 wt, although the reduction was seen in (almost) every experiment. It is therefore safe to conclude, that any of the mutations we introduced into the AH, reduces but does not eliminate fatty acid transfer to M2. Even after removal of all residues beyond Cys50, M2 is still about 50% acylated compared to M2 wt. This suggests that the amino acids preceding the acylation site also affect acylation.

### The transmembrane region effects acylation of M2

Replacing a kink-inducing glycine in the middle of the transmembrane helix with an alanine increased palmitoylation, which was statistically significant in the context of the M2 1–50 mutant (Fig. 7). Glycine is not one of the residues identified by MD simulations to contact DHHC20, but it does cause a small kink in the middle of the helix^33^. We therefore suggest that the altered conformation of the transmembrane region presents the cysteine in a more favourable way to the active site of the acyltransferase. Note also that the acylated cysteine is always at position 50 in each variant of M2, suggesting that its position plays an important role. This is in contrast to the acylated cysteines in the cytoplasmic domain of the spike proteins of corona and influenza viruses, whose positions have shifted during viral evolution^44^.

### Mutations in the helix of M2 do not prevent binding to DHHC20

Using co-immunoprecipitation assays we investigated whether M2 stably interacts with DHHC20 and whether this interaction is affected by the mutations induced (Fig. 8). If we assume that the binding of M2 to DHHC20 represents the formation of an enzyme–substrate complex, then this is reduced only for M2 AH-5 which at least partly explains its reduced acylation level. The interaction of two other mutants M2-1P and M2-3P with DHHC20 is basically undiminished and two mutants, M2-AH-4 and M2 1–50 bind even stronger to DHHC20, although acylation was significantly reduced. We conclude that the fatty acid transfer is affected in these mutants, perhaps because binding happens in an unfavourable manner.

This assumption is supported by molecular dynamics simulations of M2 mutants with DHHC20. The acylation site of truncated M2 1–50 moves away from the catalytic centre of the enzyme but the protein remains associated with TM1 and helps to explain why DHHC20 can be co-precipitated with M2 1–50 but acylation is reduced. Likewise, MD of the mutant M2-1P (Ile51Pro) showed that the insertion of the Pro does not only destroy the part of the helix upstream of Cys50, but also decreases the angle between the helix and the transmembrane region. This does not change the distance between Cys156 in DHHC20 and Cys50 in M2 but causes “misalignment” of the helix with the catalytic centre of DHHC20 as discussed below.

### Molecular dynamics simulation of the DHHC20 M2 complex: possible implications for the catalytic mechanism of the acylation reaction

The molecular dynamics simulation revealed that several residues of M2 wt are in the immediate vicinity to functionally important amino acids in DHHC20. Ile39 at the cytosolic end of the transmembrane region of M2, interacts with the Trp158 which is located near the opening of the hydrophobic cavity (Fig. 9B). The side chain of Trp158 was found in another molecular dynamics study to adopt two conformations, an open and a closed state, and it was therefore proposed that Trp158 acts as a gate for the hydrophobic cavity^41^. Thus, one can speculate that this interaction opens the gate for fatty acid transfer to M2.

Phe47 at the beginning of the amphiphilic helix is in close proximity to His154 whereas Cys50 contacts both Asp153 and His154 of the _153_DHHC_156_ motif (Fig. 9B). One might speculate that His154 of DHHC20 deprotonates Cys50 to form the reactive thiolate which then can attack the thioester linkage between Cys156 and the fatty acid. In accordance, mutation of this histidine in the DHHC motif of the yeast enzyme Erf2 abolished palmitate transfer to the Ras2 substrate^9^. However, to act as a base His154 must be polarized by the neighbouring Asp153. The crystal structure of the autoacylated form of DHHC20 shows that Asp153 interacts with Thr241 of the TTXE motif hindering its function for the deprotonation of His154. In addition, His154 interacts with the carboxylate of the palmitoylation inhibitor 2-bromopalmitate, which is covalently bound to Cys156 via C2 (Supplementary Fig. 8).

MD simulations showed that Arg53 of M2 contacts Thr241 of the TTXE motif and the two adjacent amino acids Ser244 and Phe245 (Fig. 9C). It is thus tempting to speculate that this interaction could compete with the intramolecular interaction of Thr241 with Asp153 observed in the autoacylated form of DHHC20. This would release the carboxyl group of Asp153 to deprotonate His 154 and thus initiate the fatty acid transfer from DHHC20 to M2.

MD simulations of mutant Arg53Ala corroborate this speculation by examining the distance between the sidechain oxygen of Thr241 and the C$\gamma$ of Asp153 in both the wt and mutant as shown in Supplementary Fig. 7. While the contact of residue 53 to Thr241 of the TTXE motif is maintained, Ala (unlike Arg) cannot form a hydrophilic interaction with Thr241 and thus the Arg53Ala mutant has a distance distribution shifted lower that favours an interaction with Asp153. This then prevents the proposed shifting of hydrogen bonds between the TTXE and DHHC motif. Likewise, exchange of Ile51 by Pro (and to a lesser extent by Ala) decreases the angle between the TM and the amphiphilic helix of M2. This altered geometry causes Phe47 to lose contact with His154, which needs to be deprotonated to act as a base. These altered interactions might explain the reduced acylation of mutant M2 1P although binding to DHHC20 is not affected.

In summary, we have shown that M2 requires an amphiphilic helix for efficient acylation. Based on MD simulations we speculate that specific interactions of amino acids in the helix of M2 with the DHHC and TTXE motif of DHHC20 initiate the fatty acid transfer. However, since several of the mutants contain exchanges in residues that do not contact DHHC20, general biophysical features of the helix, such as charge, hydrophobicity and hydrophobic moment play a crucial role for efficient acylation. Evidence of this is seen as M2 with mutations in the amphiphilic helix can still bind to DHHC20 but the subsequent step, the transfer of the fatty acid to M2, is impaired.

Cellular proteins are also acylated by DHHC20 on a cysteine at one end of an amphiphilic helix, usually localized near a membrane-spanning region^16–18,45^. There is no sequence similarity between these helices, suggesting that DHHC20 can acylate a variety of helical structures if they fit geometrically into the depression underneath the membrane-spanning region and have the correct biophysical properties. DHHC2, 8 and 15, which are also involved in acylation of M2^24^ contain the same cavity, and the superposition of their structures shows that the DHHC and TTXE motifs are perfectly aligned (data not shown) suggesting that the same considerations also apply for these DHHCs.

DHHC20 efficiently acylates typical viral spike proteins that do not have an amphiphilic helix in their cytoplasmic tail^24,46^ and hence the enzyme might recognize other features in substrate proteins. It is not uncommon that the same DHHC exhibits more than one mode of enzyme–substrate interactions^47^. This is mediated by interactions between transmembrane regions, since the M2 1–50 mutant, which lacks most of the amino acids proposed to initiate fatty acid transfer, is still acylated. However, it is also conceivable that the basal acylation is due to a non-enzymatic fatty acid transfer. This has been described for purified proteins in the presence of Pal-CoA, but only at basic pH to deprotonate the cysteine. However, it also occurs in membranes at physiological pH, albeit less efficiently than the corresponding enzymatic reaction^48^. The stable interaction with DHHC20 could lower the pKa of Cys50 in M2, which then spontaneously attacks the carbonyl carbon of Pal-CoA^49^. Purification of substrate and DHHC enzymes and in vitro reconstitution of the acylating activity are required to clarify these open questions^50^.

## Materials and methods

### Cell lines, genes and plasmids

HEK293T cells and BHK21 cells, initially obtained from the American Type Culture Collection (ATCC number CRL-11268 and CCL-10, respectively) were grown in Dulbecco’s modified Eagle’s medium (DMEM, PAN Biotech) supplemented with 10% fetal bovine serum, 100 U/ml penicillin, and 100 µg/ml streptomycin at 37 °C with 5% CO2. 293 T cells were used to express the protein and test the S-acylation level. BHK21 cells were used for the immunofluorescence experiment. The M2 and M2 1–50 from human Influenza A virus A/WSN/33 (H1N1) (GenBank: CY034133.1) and RFP was cloned from LV-RFP (addgene: #26001with XhoI and BgIII (NEB) into the PCAGGS expression plasmids. M2 mutants were generated with quick change site-directed mutagenesis method with S7 Fusion Polymerase from Mobidiag. The clones of the RFP-AH and RFP-AH-C50S were made using restriction cloning with XhoI and BgIII (NEB) into the PCAGGS, the reverse primer encode the sequence of AH and AH-C50S of M2. Sequences of the primers are listed in the supplementary materials. The human DHHC20 gene equipped with a C-terminal myc-tag and cloned into pcDNA3.1 was provided by the Fukata lab^51^.

### Antibodies and reagents

Primary Antibodies: Monoclonal anti-M2 antibody 14C2 from mice directed against the ectodomain (Santa Cruz Biotechnology, sc-32238). Purified polyclonal Anti-Flotillin-2 antibody from mice (BD, 610383). polyclonal anti-RFP antibody from rabbit (abcam, ab62341), monoclonal anti-myc-tag antibody from rabbit (Proteintech, 10828-1-AP), monoclonal anti-GM130 cis-Golgi Marker antibody from rabbit (abcam, ab52649).

Secondary Antibody: Anti-rabbit IgG VHH single domain antibody coupled to HRP (abcam, Ab191866). anti-mouse IgG (H + L) coupled to HRP from goat (Biorad, 1706516). anti-mouse IgG (H + L) coupled to Alexa 488 from goat (Thermo Fisher Scientific, #A28175). anti-Rabbit IgG (H + L) coupled to Alexa 488 from goat (Thermo Fisher Scientific, #A-11008), anti-Rabbit IgG (H + L) coupled to Alexa 568 from goat (Thermo Fisher Scientific, #A-11011);

Reagents: Protein inhibitor (Roche/Merk, 11873580001) was used in all the protein experiments except the membrane separation experiment, where the inhibitor in the kit was used.

NP40 lysis buffer (Thermo Scientific, 85124), Pierce ECLplus reagent (Thermofisher, #32132), Thiopropyl Agarose (Creative biomart, Thio-001A), methyl methanethiosulfonate (MMTS, Sigma, 208795), Tris (2-carboxyethyl) phosphine (TCEP, Carl Roth, HN95.2), ProLong Glass antifade mounting medium (Thermo Fisher, P36984), lipofectamine 3000 transfection reagent (ThermoFisher, L3000015),the subcellular protein fractionation kit for cultured cells (ThermoFisher, 78840), DAPI (4’,6-diamidino-2-phenylindole, ThermoFisher, 62247), Protein-G Sepharose 4 Fast Flow(GE Healthcare, GE17-0618-01), S7 Fusion Polymerase (Mobidiag, MD-S7-500).

### Membrane separation experiment

3 µg plasmids encoding RFP, RFP-AH or RFP-AH-C50S were transfected into 293 T cells using lipo3000 transfection reagent. After 24 h, the subcellular protein fractionation kit for cultured cells was used to separate cells into cytoplasm and membranes. All the buffers were prepared with protein inhibitors from the kit in advance. Cells (1 × 10^6^), PBS-washed and pelleted in a microfuge (500×*g*, 5 min), were incubated with 150ul of the cytoplasmic extraction buffer CEB at 4 °C for 10 min with gentle mixing. This buffer permeabilizes the plasma membrane and releases the cytosol. The opened cells are pelleted (500×*g*, 5 min) and the supernatant (cytosol) is removed. 150 ul membrane extraction buffer MEB was added to the pellet, first vortex the tube for 5 s and incubate tube at 4 °C for 10 min with gentle mixing This dissolves all membranes with the exception of the nuclear membrane. Centrifugation (3000×*g* for 5 min) pellets the nuclei, the supernatant contains extracts from the plasma membrane, mitochondria, and ER/Golgi membranes. 10% of the cytosol preparation and 20% of the membranes were analysed by western blotting using anti-RFP antibodies (1:1000) and secondary anti-rabbit IgG VHH single domain antibody (1:3000) as described below.

### Co-Immunoprecipitation experiments

Plasmids (1.5 µg) encoding M2-wt or M2 mutants were co-transfected with the plasmid (1.5 µg) encoding human DHHC20 fused at its cytosolic C-terminus to a myc tag into 293 T cells grown in 6-well plates. 24 h after transfection cells were lysed with 250 µl NP40 lysis buffer (final concentration 0.5%) diluted in IP buffer (500 mM Tris–HCl, 20 mM EDTA, 30 mM sodium pyrophosphate decahydrate, 10 mM sodium fluoride, 1 mM sodium orthovanadate, 2 mM benzamidine, 1 mM PMSF, 1 mM NEM) with protein inhibitor for 1 h on ice. Cell debris were removed by centrifugation for 10 min at 10,000 rpm in a table top centrifuge. To determine the expression level (input control) 10% of the resulting supernatant was removed and subjected to western-blotting with either anti-M2 monoclonal antibody from mice (1:4000) or anti-myc-tag polyclonal antibody from rabbit (1:2000).

1 µl of the M2 antibody (diluted in 200ul 3% BSA buffer) was added to the remaining supernatant and incubated for 1 h at 4 °C with shaking. Later 30 µl of protein-G Sepharose 4 Fast Flow (GE Healthcare, prepared according to manufacturer instructions) were added to the samples and incubated at 4 °C with shaking overnight. Samples were then centrifuged for 5 min at 5000 rpm and the supernatant was removed. After three times washing with IP buffer, the pellet was dissolved in 40 µl of 2X SDS-PAGE loading buffer and subjected to western-blotting, first with anti-myc-tag antibody and secondly with anti-M2 antibody using the appropriate secondary antibodies as described below. The relative amount of DHHC20-myc coprecipitated with the M2 antibodies was calculated using the band density of the DHHC20-myc band divided by the density of the M2 band visualized on the same membrane.

### Acyl-resin assisted capture (Acyl-RAC)

Protein S-acylation was analyzed by the Acyl-RAC assay^52^. Transfected 293 T cells were washed with PBS twice, and each well of the 6-well plate was lysed in 500 µl buffer A (0.5% Triton-X100, 25 mM HEPES (pH 7.4), 25 mM NaCl, 1 mM EDTA, and protease inhibitor cocktail). Aliquots of the sample was used to check for total protein expression by western blotting. Disulfide bonds were reduced by adding Tris (2-carboxyethyl) phosphine (TCEP) to a final concentration of 20 mM and incubated at RT for 1 h. Free SH-groups were then blocked by adding methyl methanethiosulfonate (MMTS), dissolved in 100 mM HEPES, 1 mM EDTA, 87.5 mM SDS to a final concentration of 1.5% (v/v) and incubated for 4 h at 40 °C. 3 volumes of ice-cold 100% acetone were added to the cell lysate and incubated at − 20 °C overnight. Precipitated proteins were pelleted at 5000×*g* for 15 min at 4 °C. Pelleted proteins were washed five times with 70% (v/v) acetone, air-dried, and then re-suspended in 1 ml binding buffer (100 mM HEPES, 1 mM EDTA, 35 mM SDS) with protein inhibitor. An aliquot, adjusted according to the expression level was removed and served as input. Another aliquot of the sample, also adjusted according to the expression level, was treated with hydroxylamine (0.5 M final concentration, added from a 2 M hydroxylamine stock adjusted to pH 7.4) to cleave thioester bonds. The same volume of the sample was treated with 0.5 M Tris–HCl (pH 7.4). 40 µl Thiopropyl Agarose, which were washed twice in binding buffer in 1000 rpm 10 min before use was added at the same time to capture free SH-groups. Samples were incubated with beads overnight at room temperature on a rotating wheel. The beads were then washed 4 times in binding buffer and proteins were eluted from the beads with 2 × reducing SDS-PAGE sample buffer for 10 min at 95 °C. Samples were subjected to western blot as described below.

### SDS-PAGE and western blot

After sodium dodecyl sulphate–polyacrylamide gel electrophoresis (SDS-PAGE) in 12% gels, wet electroblotting (100 V for 1 h) was used to transfer proteins onto polyvinylidene difluoride (PVDF) membranes (GE Healthcare). After blocking (blocking solution: 5% skim milk powder in phosphate-buffered saline (PBS) containing 0.1% Tween-20 (PBST)) for 1 h at room temperature, membranes were incubated with the indicated primary antibody diluted in 3% BSA in PBST overnight at 4 °C. The following commercial antibodies were used anti-M2 (1:1000), Purified Mouse Anti-Flotillin-2 (1:1000). Anti-RFP antibody (1:1000), anti myc-tag antibody (1:2000). After washing 3 times for 10 min with PBST, membranes were incubated with horseradish peroxidase-coupled secondary antibody diluted in 3% BSA for 1 h at room temperature. The following commercial secondary antibodies were used anti-rabbit IgG VHH Single Domain (1:3000 to 1:5000) and goat anti-mouse IgG (H + L), (1:1000 to 1:3000). After washing three times with PBST, signals were detected by chemiluminescence using the Pierce ECLplus reagent and a Fusion SL camera system (Peqlab, Erlangen, Germany). The density of bands was quantified with Image J software as described in the figure legends. All uncropped membranes are shown in the Supplementary Information file.

### Confocal microscopy

#### Co-localization of M2 with cis-golgi marker GM130

BHK21 cells were seeded at 50% confluency one day before transfection on glass coverslips in 24-well cell culture plates. Cells were transfected with 500 ng plasmids encoding M2 (wt or mutants) using lipofectamine 3000 transfection reagent according to manufacturer´s instructions. 24 h post transfection, cells were fixed with 4% paraformaldehyde (PFA) for 20 min at room temperature, washed two times with PBS, permeabilized with Triton X-100 (0.1% in PBS) for 10 min and again washed twice with PBS. To coat non-specific protein binding sites, cells were incubated with 3% bovine serum albumin (BSA in PBS containing 0.1% Tween-20) for 1 h at room temperature. BSA was removed and cells were incubated with the first antibody for 1 h at room temperature, the anti-M2 antibodies (1:1000 dilution in 3%BSA) was used. After washing with PBS three times for 5 min, cells were incubated with fluorescent anti-mouse Alexa flour 488 from goat (1:1000). Cells were washed 3 times for 5 min with PBS and then incubated with anti-GM130 antibody (1:200) for one hour. Cells were then washed 3 × for 5 min with PBS and then treated for 1 h protected from light at RT, cells were stained simultaneously with anti-rabbit Alexa 568 from goat (1:1000) for 1 h at room temperature. Cells were washed 3 times for 5 min with PBS.

#### Co-localization of M2 with DHHC20

To detect DHHC20-myc and M2, 300 ng of each plasmid were co-transfected in BHK21 cells. M2 was visualized as described above. Then anti myc-tag rabbit antibody (1:1000) followed by anti-rabbit Alexa fluor 568 from goat antibody (1:1000) were used to detect DHHC20-myc location.

Cells were subsequently stained with DAPI (4′,6-diamidino-2-phenylindole, 1:1000 dilution in BSA) for 10 min at RT to visualize nuclei. Cells were washed three times with PBS and coverslips were mounted on glass slides with ProLong Glass antifade mounting medium and allowed to cure in a dark place overnight.

#### Visualization of RFP

500 ng plasmids encoding RFP (wt or fused to the amphiphilic helix of M2) were transfected into BHK21 cells. Cells were fixed and mounted on glass slides as described above.

Cells were illuminated via laser lines at 405 nm (DAPI), 488 nm (Alex Fluor 488) and 568 nm (Alexa Fluor 568 and RFP protein) and visualized with the VisiScope confocal FRAP System (VisiTron Systems GmbH), equipped with iXon Ultra 888 EMCCD camera using the 100X objective. The images were then processed using Fiji software. Co-localization of M2/M2 mutates with the cis-Golgi marker was quantified from at least 40 cells with the Pearson's correlation coefficient method using the JACoP plugin of the Fiji software.

### Bioinformatic procedures

The helical wheel plots were made with Heliquest (https://heliquest.ipmc.cnrs.fr/) using the amphiphilic helix (residues 47 to 61) of the M2 sequence (ACF54600.1). Protein structures were analysed with PyMOL (Molecular Graphics System, Version 2.0 Schrödinger, LLC, https://pymol.org/2/).

### Molecular modelling of the M2 DHHC 20 interaction

Starting from an available structure of the M2 (PDB ID: 2LOJ) the mutations Ser50Cys, Phe54Arg, Glu56Lys, and His57Tyr were performed to match the M2 used in the experimental assay. The starting structure for DHHC (PDB ID: 6BML) was selected taking only chain A, the bound Zn^2+^ ions, and the palmitoyl acid group (PAL)^33,53^. The docking software Haddock [https://wenmr.science.uu.nl/haddock2.4/] was used to obtain several poses of the complex. The two pdb structures 6BML (autoacylated from of DHHC20) and 2L0J (M2) were input to Haddock with residue 50 of M2 selected as active with partner 154 of DHHC. The output of this was examined and the most stable cluster with aligned transmembrane sections was taken as the input sequences are not initially membrane bound. This corresponded to a cluster of 7 structures with an average Haddock score of -115.5 and RMSD to the lowest predicted energy structure of 6.6 $\pm$ 0.3 Å from which we select the top ranked structure. Further input scripts and parameters to Haddock can be found in the link in the data availability section. This structure was fed into CharmmGUI^54^ and inserted into a POPC membrane (105/100 in upper/leaflet respectively) before being solvated and ionized to 150 mM using NaCl. Using this output as the starting conformation, minimization for 1000 conjugate gradient steps followed by a temperature equilibration simulation where a Langevin thermostat with a damping coefficient of 1 $p{s}^{-1}$ was increased by 1 K every 5 ps until 298 K was reached, the entire time keeping non-solvent atoms constrained to their initial positions. From here the structure is passed from NPT (maintained at 1 atm with a Langevin piston period and decay of 100 and 50 timesteps respectively) to NVT to NPT for rounds of 2 ns to allow for solvent relaxation and further pressure and volume equilibration. Production runs were performed for 200 ns at constant volume. During all simulations the PAL is kept ”bound” to the DHHC with a 1-sided harmonic wall with upper boundary at 4 Å and a strength of 25 kcal/mol/Å and conformational constraint on the Zn^2+^ ions to keep them in their binding pocket. Mutation structures for a Ile51Ala and Ile51Pro were generated using the VMD plugin Mutator. As single point mutations are unlikely to affect the docking structure, the same equilibrated structure is used as before. The Ile51Ala and Ile51Pro simulations were run in a straightforward fashion for 200 ns. All MD simulations were carried out using NAMD version 2.14^55^ and the CHARMM36 force field^56^. Electrostatic interactions were calculated using PME, with a cutoff of 10 Å, switching distance of 8 Å, pair list distance of 12 Å, and an integration time step of 2 fs. Rigid bonds for hydrogen atoms were used and snapshots were saved every 2000 timesteps. A sample configuration file can be found in the link in the Data Availability section.

In addition to the single point mutations listed above, we also construct a thermodynamic cycle for a Arg53Ala mutation to measure the change in binding free energy upon mutation^57^. See Supplementary Fig. 7C for a cartoon representation or Fig S8 of corresponding reference for a pictorial description.

$$\Delta {G}_{WT\to Mut}^{M2}+\Delta {G}_{Mut}^{Bind}=\Delta {G}_{WT}^{Bind}+\Delta {G}_{WT\to Mut}^{complex},$$

where $\Delta {G}_{WT\to Mut}^{M2}$ &$\Delta {G}_{WT\to Mut}^{complex}$ measures the change in free energy under mutation for the M2 alone and M2/DHHC complex respectively while $\Delta {G}_{WT}^{Bind}$ & $\Delta {G}_{Mut}^{Bind}$ measures the change in free energy upon binding M2 to DHHC to form the complex for the wt and mutant respectively. For this cycle we observe $\Delta {G}_{WT\to Mut}^{M2}$ and $\Delta {G}_{WT\to Mut}^{complex}$ so a negative $\Delta \Delta G$ ($\Delta {G}_{WT\to Mut}^{M2}-\Delta {G}_{WT\to Mut}^{complex}$) indicates that the wt binds more stably. To perform the alchemical mutagenesis, we use the dual topology approach in NAMD^55^ that uses a switching parameter, varied between the values of 0 and 1, in the Hamiltonian to smoothly connect the wt and Arg53Ala mutant. In this instance the switching parameter is modified from 0 to 1 over a course of 20 equally spaced steps where 0 represent the wt and 1 the Arg53Ala mutant. When undergoing this side chain atom transformation, there is a change in the total net charge. To account for this a Cl^-^ ion is placed under the influence of the switching parameter and constrained to reside in the bulk solution to ensure total charge neutrality. At each switching parameter step the structure is minimized over 100 steps and run for 10 ps before a 12.5 ns production run. We vary the switching parameter in both the forward and backward direction to use the more efficient Bennett Acceptance ratio^58^ in estimating the free energy. We perform 2 sets of simulations starting from different initial conformations and reported values are the mean and standard error. Convergence of this quantity by measure of the relative standard deviation, a measure that the first two moments have converged, is shown in Supplementary Fig. 7D.

To measure different localization sites, we perform what is referred to in the later parts of the text as contact analysis. This is performed by analysing the amino acid pairs that have an atom distance < 4 Å and we only report values that are present for more than 30% of the simulation time after an initial 40 ns of further equilibration, during which time no constraints other than the one placed on the Zn^2+^ and PAL residues are placed.

### Supplementary Information

Supplementary Information.

## Data Availability

Most data generated or analysed during this study are included in this published article and its supplementary information files. The data and analysis scripts of the MD simulations are uploaded on Zenodo (https://doi.org/10.5281/zenodo.8305289).
