# Supplementary material for: The role of an amphiphilic helix and transmembrane region in the efficient acylation of the M2 protein from influenza virus
Source: Sci Rep. 2023 Nov 2;13:18928. doi: 10.1038/s41598-023-45945-z (PMC10622425; doi:10.1038/s41598-023-45945-z)
Supplement: Supplementary file 1 — Supplementary Information. [file 41598_2023_45945_MOESM1_ESM.pdf]

Supplementary files

The role of an amphiphilic helix and transmembrane region in the efficient acylation of the M2 protein from Influenza virus

Xiaorong Meng[1], Clark Templeton[2], Cecilia Clementi[2], Michael Veit[1#]
1 Institute of Virology, Veterinary Faculty, Free University Berlin, Germany
2 Theoretical and Computational Biophysics, Department of Physics, Free University Berlin, Germany

Supplementary Figure 1

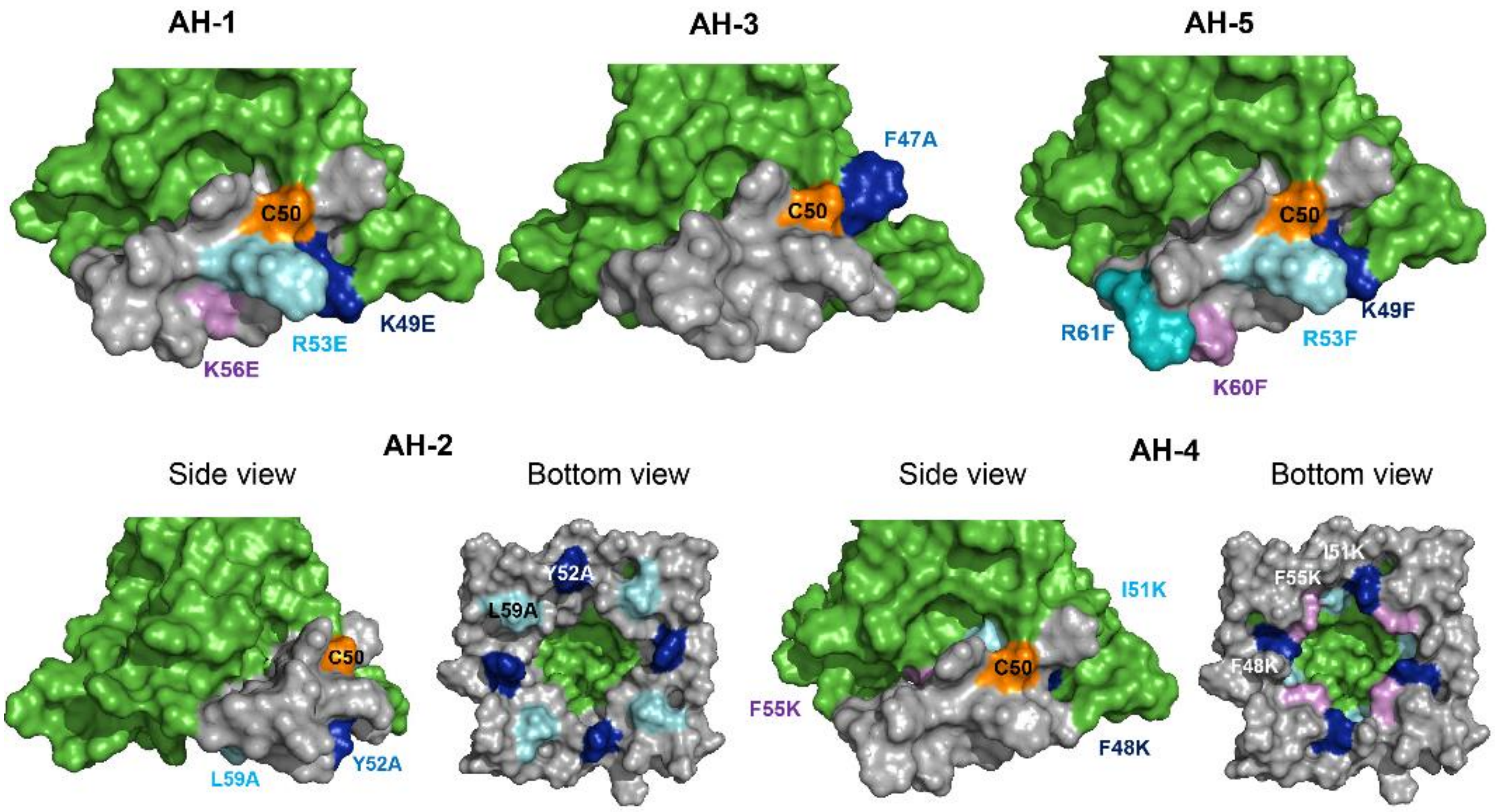

Supplementary figure 1: Location of the amino acids exchanged in the amphiphilic helix of M2
Surface representation of the NMR structure of the M2 tetramer. Amino acids of one amphiphilic helix are coloured grey, the acylation site in orange and the amino acids exchanged in AH-1, AH-2, AH-3, AH-4 and AH-5 are coloured in dark or light blue or in magenta.

Supplementary Figure 2

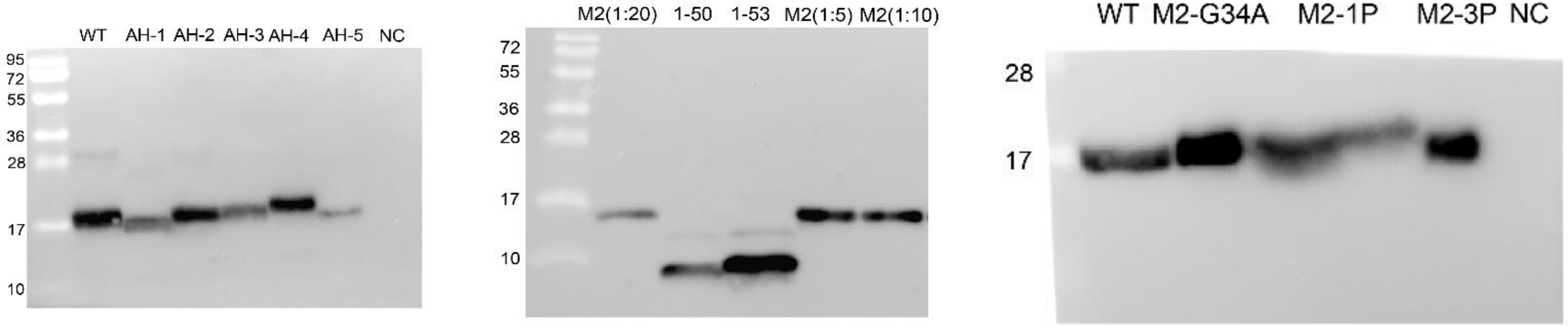

Supplementary figure 2: Comparison of the expression levels of M2 wt and the indicated mutants. M2 wt and the specified M2 mutants were expressed in 293T cells. Cells were lysed and an aliquot was subjected to blotting with antibodies against M2. The numbers in brackets indicate the dilution of the lysate before SDS-PAGE. NC: untransfected cells.

Supplementary Figure 3

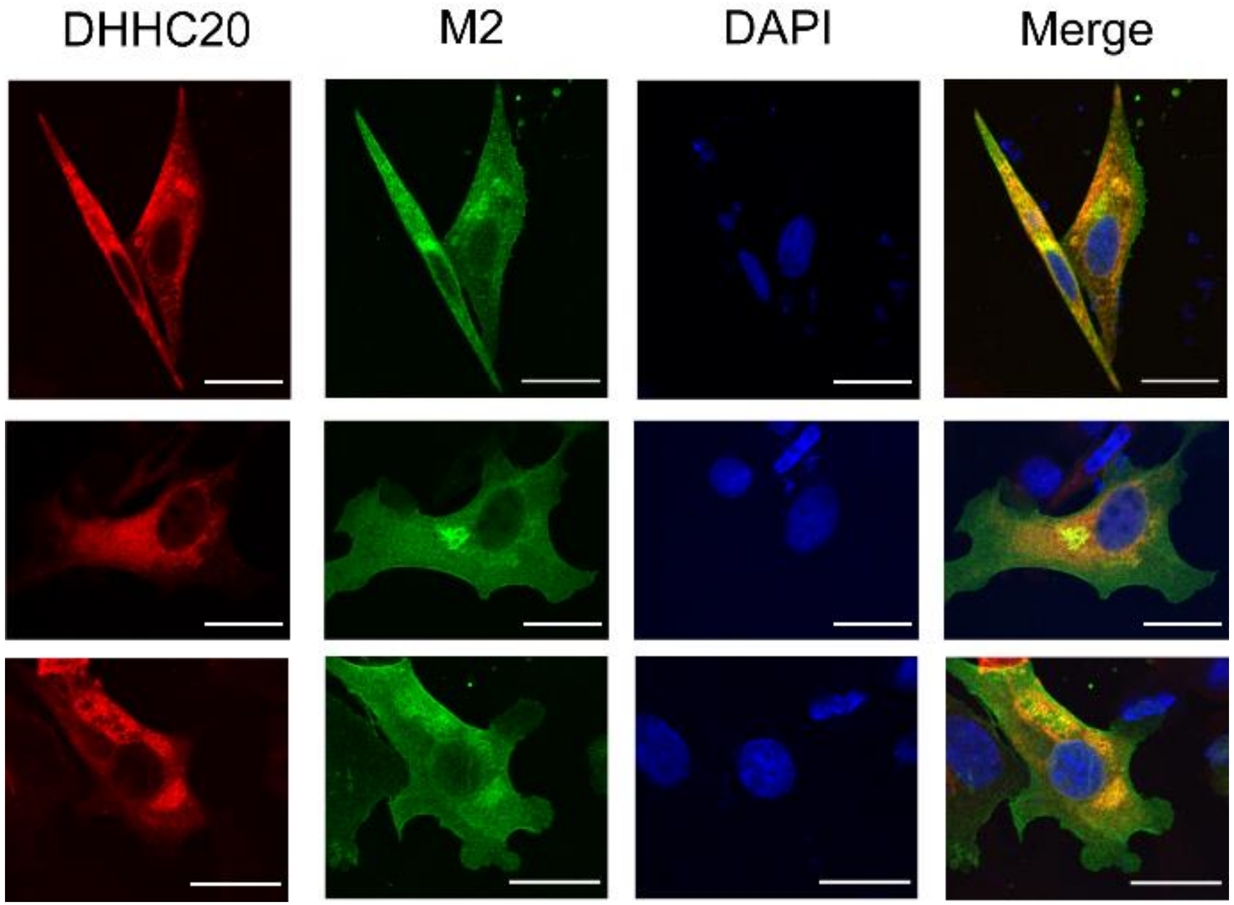

Supplementary figure 3: Colocalization of DHHC20 with M2
M2 wt and DHHC20-myc were expressed in BHK21 cells, which were fixed, permeabilized and stained with anti-myc and anti-M2 antibodies. The scale bar is 20 µm.

Supplementary Figure 4

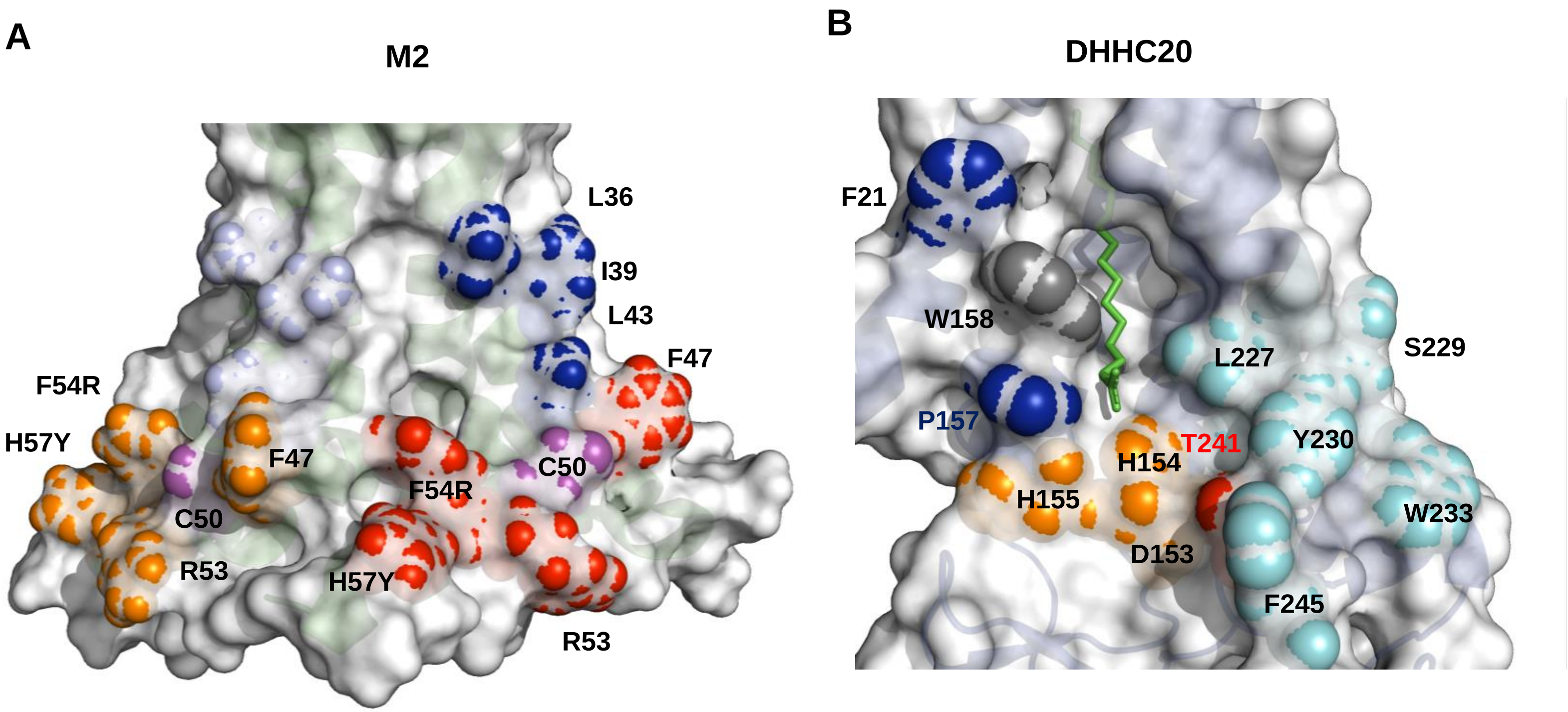

Supplementary figure 4:

(A) Surface representation of the NMR structure of a M2 tetramer (pdb file 2L0J) highlighting the amino acids in close proximity to DHHC20. Blue and light blue spheres: amino acids in the transmembrane region, red and orange spheres in the amphiphilic helix. Magenta sphere: acylation site. Note that two residues differ between the NMR and modelled structure, indicated by the two letters in the labels. First letter: aa in the NMR structure, second letter: aa in the modelled structure.

(B) Surface representation of DHHC20 (pdb file 6bml) highlighting the amino acids in close proximity to M2. Residues in TM1 and TM4 of DHHC20 that interact with the TM of M2 are shown as blue and cyan spheres, respectively. Amino acids of the DHHC motif are shown as orange spheres. Grey sphere: W158, black sphere: T241 of the TTXE motive. Green stick: Fatty acid.

Supplementary Figure 5

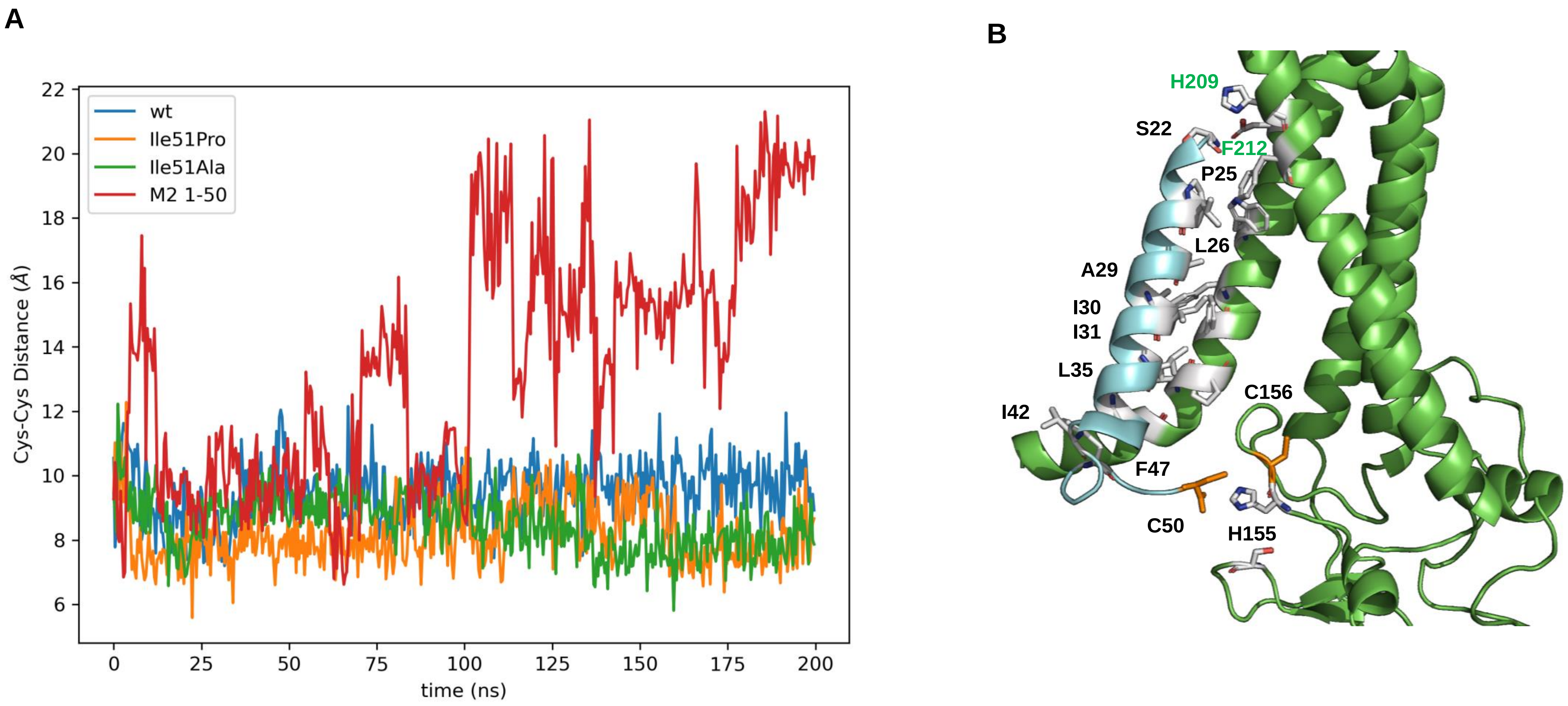

Supplementary figure 5

(A) Measured distance between sulfur groups of M2-Cys50 and DHHC-Cys156 as a function of trajectory length. The legend indicates the particular mutation performed.

(B) Snapshot of the MD simulation with DHHC20 (green) and M2 1-15 (cyan). Contacting residues are shown as white sticks, Cys50 in M2 and Cys156 in DHHC20 as orange sticks.

Supplementary Figure 6

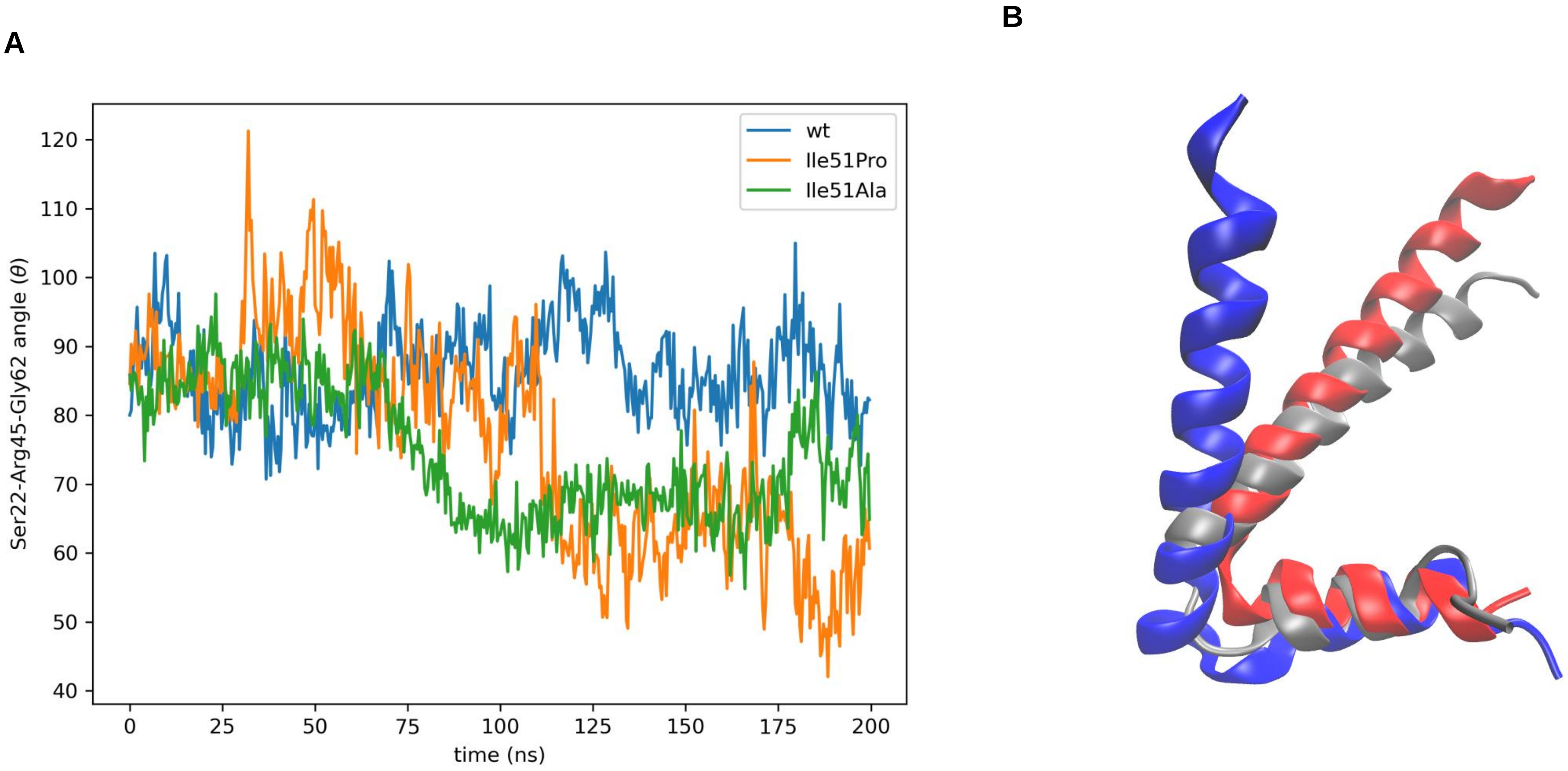

Supplementary figure 6

(A) Time trace of the angle formed by the Cα atoms of Ser22 - Arg45 – Gly62 of M2. The legend again indicates the mutation performed.

(B) Snapshot of MD simulation of rmsd-aligned M2 residue with wt (blue), Ile51Ala (red), and blue (Ile51Pro).

Supplementary Figure 7

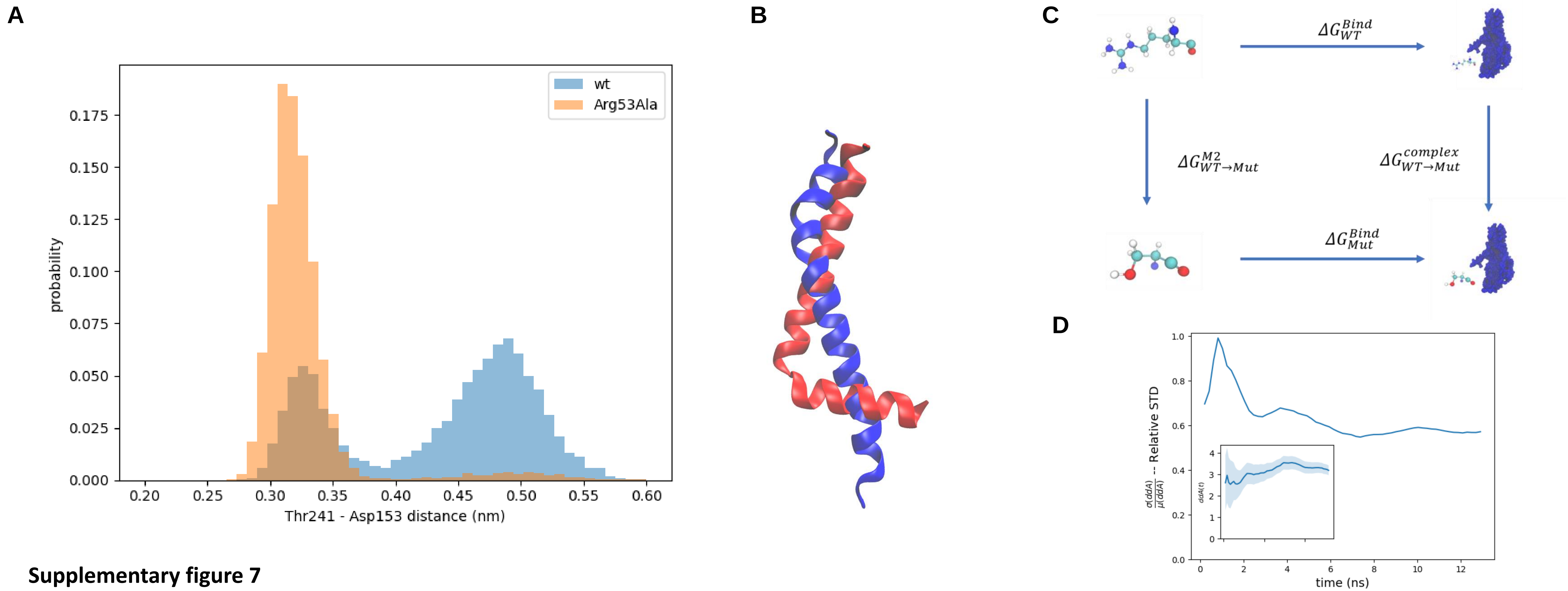

Supplementary figure 7

(A) Probability of the distance distribution between the oxygen atom on Thr241 and the Cγ of Asp153 of DHHC. The wt (switching parameter = 0) is shown in blue and the Arg53Ala mutant (switching parameter = 1).

(B) RMSD aligned M2 only simulations of the wt (blue) and Arg53Ala (red).

(C) Scheme for calculation of relative binding free energies using alchemical substitutions. The four panels show WT M2 bound to DHHC (top right), the Arg53Ala mutant M2 bound to DHHC (bottom right), WT M2 in solution (top left), and the mutant in solution (bottom left). Horizontal arrows correspond to free energy differences associated with the formation of the complex, which can be measured experimentally. Vertical arrows refer to free energy differences in the complex and with the RBD alone which are computed numerically via alchemical methods.

(D) The relative standard deviation ddA over simulation time for alchemical simulations. Convergence of this value implies that at least the first two moments have converged and seems to appear by ~10ns. The inset shows the raw ddA with error between the two replicas given by the shaded region.

Supplementary Figure 8

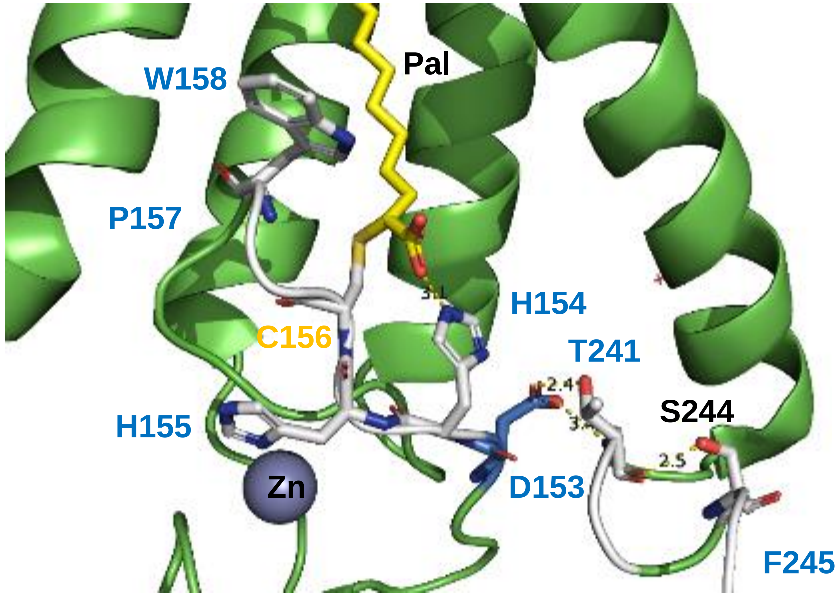

Supplementary figure 8: Structure of the autoacylated form of DHHC20
The amino acids in DHHC20 that interact with the helix of M2 are labeled in blue. Created with PyMol from pdb-file 6BML.

Supplementary Figure 9

Quantification of co-localization
shown in figure 4C

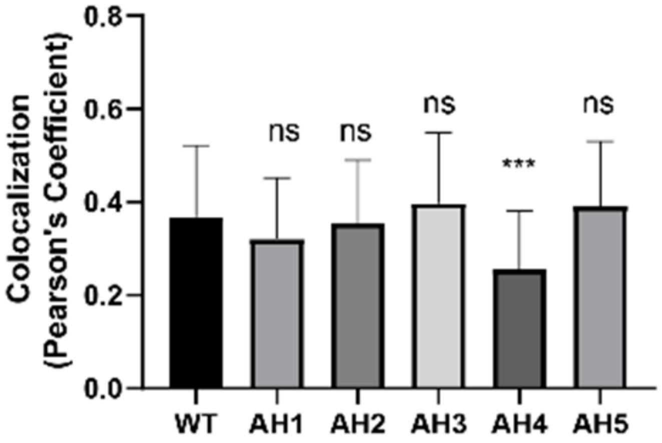

Quantification of co-localization
shown in figure 5B

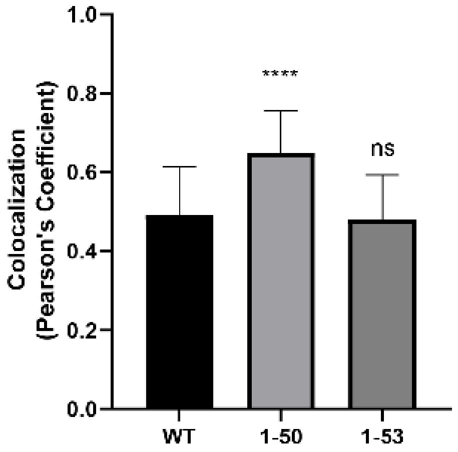

Quantification of co-localization
shown in figure 6B

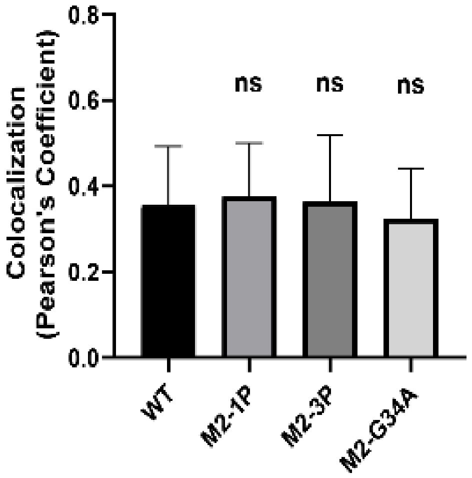

Quantification of co-localization
shown in figure 7B

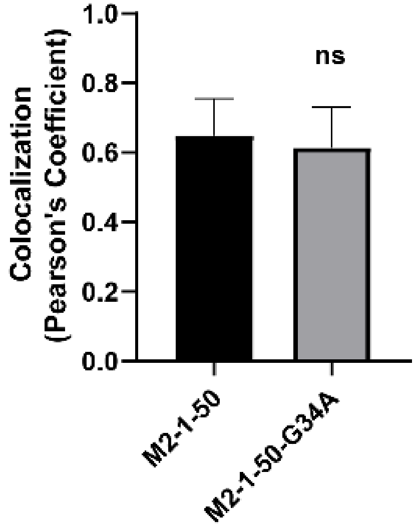

Supplementary figure 9: Quantification of co-localization of M2 with cis-Golgi marker GM130.
Co-localization from at least 50 cells was quantified with Pearson's correlation coefficient method using the JACoP plugin of the Fiji software. One-way ANOVA followed by multiple comparison Tukey test was applied for statistical analysis. ***, P < 0.001, ****, P <0.0001

Supplementary table 1

Name	Sequence	Acylation	Binding	Features AH
wt	FFKCIYRRFKYGLKR	100%	+	
AH-1	FF**E**CIY**E**RF**E**YGLKR	62%	n.a.	No net charge
AH-2	FFKCI**A**RRFKYG**A**KR	49%	n.a.	<H> ↓
AH-3	**A**FKCIYRRFKYGLKR	74%(ns)	n.a.	<H> ↓
AH-4	F**K**KC**K**YRRKKYGLKR	54%	++	<μH>↓ <H> ↓↓
AH-5	FF**F**CIY**F**RFKYGL**FF**	30%	+/-	<μH>↓<H>↑
1P	FFKC**P**YRRFKYGLKR	30%	+	H destroyed
3P	FFKCI**PPP**FKYGLKR	82%(ns)	+	H destroyed
1-50	FFKC	54%	++	truncated
1-53	FFKCIYR	62%	n.a.	truncated

Supplementary table 1: Summary of the effects of the amino acid mutations in the amphiphilic helix of M2
"Acylation" is the normalized acylation level relative to M2 wt, "Binding" summarizes the results of the Co-IP experiments with DHHC20 and "features AH" the effect of the mutations on the biophysical property of the amphiphilic helix AH. <H>: overall hydrophobicity and <μH> hydrophobic moment of the helix. ↑and↓ indicate an increase and decrease, respectively of these features. Mutated amino acids are in red.. N.a.: not analysed, Ns: not significant.

Supplementary table 2

M2 wt		
**DHHC**	**M2**	**%**
PHE21	LEU36	0,318
PHE21	ILE39	0,318
ASP153	CYS50	0,418
HIS154	PHE47	0,714
HIS154	CYS50	0,42
HIS154	ARG54	0,606
HIS155	PHE47	0,812
PRO157	LEU43	0,482
PRO157	PHE47	0,402
TRP158	ILE39	0,514
TRP158	LEU43	0,467
PHE208	PRO25	0,398
LEU227	ARG54	0,945
SER229	TYR57	0,718
TYR230	ARG53	0,706
TYR230	ARG54	0,716
TYR230	TYR57	0,984
HIS231	ARG54	0,435
TRP233	TYR57	0,949
THR241	CYS50	0,41
THR241	ARG53	0,878
PHE245	ARG53	0,963

Supplementary table 2
Contact map of DHHC20 and M2 wt. First column lists the residues from DHHC, second from M2, third column is the percentage of time the contact is formed with the first ~40ns used for additional equilibration

Supplementary table 3

M2 1-50		
**DHHC**	**M2**	**%**
TRP7	LEU46	0.643
TRP7	PHE47	0.424
GLN11	LEU46	0.390
GLN11	PHE47	0.800
GLN11	PHE48	0.888
VAL14	ILE39	0.610
VAL14	PHE47	0.590
VAL17	LEU36	0.404
PHE21	ILE32	0.759
PHE21	ILE33	0.327
PHE21	LEU36	0.598
HIS155	CYS50	0.433
TRP158	LEU36	0.322
TRP158	LEU40	0.396
PHE208	SER23	0.492
HIS209	SER22	0.316
HIS209	PRO25	0.312
PHE212	PRO25	0.541
PHE212	LEU26	0.702
PHE212	ALA29	0.310

Supplementary table 3:
Contact map of DHHC20 and M2 1-50.

Supplementary table 4

M2 Ile51Pro				M2 Ile51Ala		
**DHHC**	**M2**	**%**		**DHHC**	**M2**	**%**
HIS154	CYS50	0,759		HIS154	PHE47	0,361
PRO157	PHE47	0,635		HIS154	CYS50	0,686
TRP158	PHE47	0,416		HIS155	PHE47	0,39
TRP197	ASP24	0,347		PRO157	PHE47	0,629
PHE215	ILE32	0,476		TRP158	LEU43	0,453
MET219	LEU36	0,449		TRP158	PHE47	0,329
LEU227	ARG54	0,971		PHE215	ILE32	0,373
SER229	TYR57	0,871		MET219	LEU36	0,365
TYR230	ARG53	0,749		SER226	ARG61	0,565
TYR230	ARG54	0,724		LEU227	ARG54	0,918
TYR230	TYR57	0,992		SER229	TYR57	0,927
HIS231	ARG54	0,312		TYR230	ARG53	0,843
TRP233	TYR57	0,886		TYR230	ARG54	0,761
THR241	ARG53	0,384		TYR230	TYR57	0,994
PHE245	ARG53	0,933		HIS231	ARG54	0,557
				TRP233	TYR57	0,778
				THR241	CYS50	0,396
				THR241	ARG53	0,594
				SER244	ARG53	0,502
				PHE245	LYS49	0,314
				PHE245	ARG53	0,857

Supplementary table 4:
Contact map of DHHC20 and M2 Ile51Pro or M2 Ile51Ala.

Supplementary table 5

M2 Arg53Ala		
**DHHC**	**M2**	**%**
VAL17	ILE39	0,755
PRO18	ILE39	0,955
PHE21	ILE32	0,779
PHE21	ILE35	0,615
PHE21	LEU36	0,888
PHE21	ILE39	0,616
PHE24	ILE32	0,568
CYS142	PHE47	0,525
ALA144	LEU46	0,504
ALA144	PHE47	0,523
CYS145	PHE47	0,386
ASP153	CYS50	0,909
HIS154	LEU43	0,626
HIS154	PHE47	0,984
HIS154	CYS50	0,998
HIS154	ILE51	0,626
HIS154	ARG54	1
HIS155	PHE47	0,97
CYS156	ARG54	0,479
PRO157	ILE39	0,544
PRO157	LEU43	1
PRO157	PHE47	0,794
TRP158	LEU36	0,962
TRP158	ILE39	0,986
TRP158	LEU40	0,921
TRP158	LEU43	0,854
PHE171	ARG54	0,738
PHE208	PRO25	0,321
PHE208	LEU26	0,494
PHE212	ALA29	0,461
SER226	ARG61	0,532
LEU227	ARG54	0,998
SER229	TYR57	0,93
SER229	ARG61	0,426
TYR230	ARG54	1
TYR230	TYR57	1
HIS231	ARG54	0,92
TRP233	TYR57	0,98
THR241	LYS49	0,331
THR241	CYS50	1
THR241	ARG54	0,815
ILE242	LYS49	0,888

ILE242	CYS50	0,908
PHE245	PHE48	0,653
PHE245	LYS49	0,982
PHE245	TYR52	0,812
TYR230	R2A53	0,99
THR241	R2A54	0,99
SER244	R2A55	0,654
PHE245	R2A56	0,875

Supplementary table 5

Contact map of DHHC20 and M2 Arg53Ala for the mutant site from the simulations with switching parameter set to 1

Uncropped western-blots

Full scan Figure 3

B

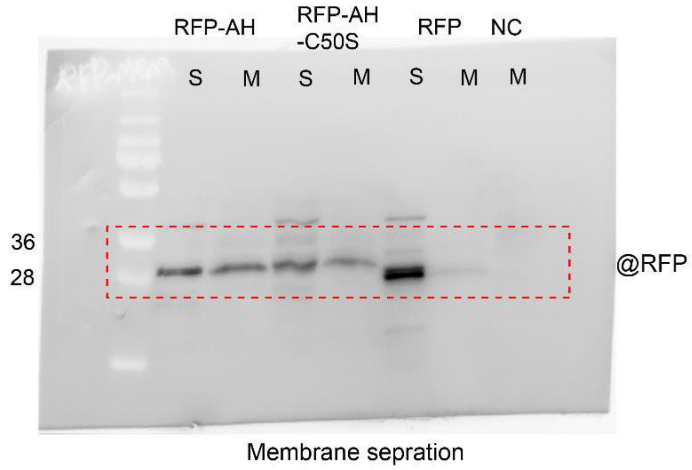

D

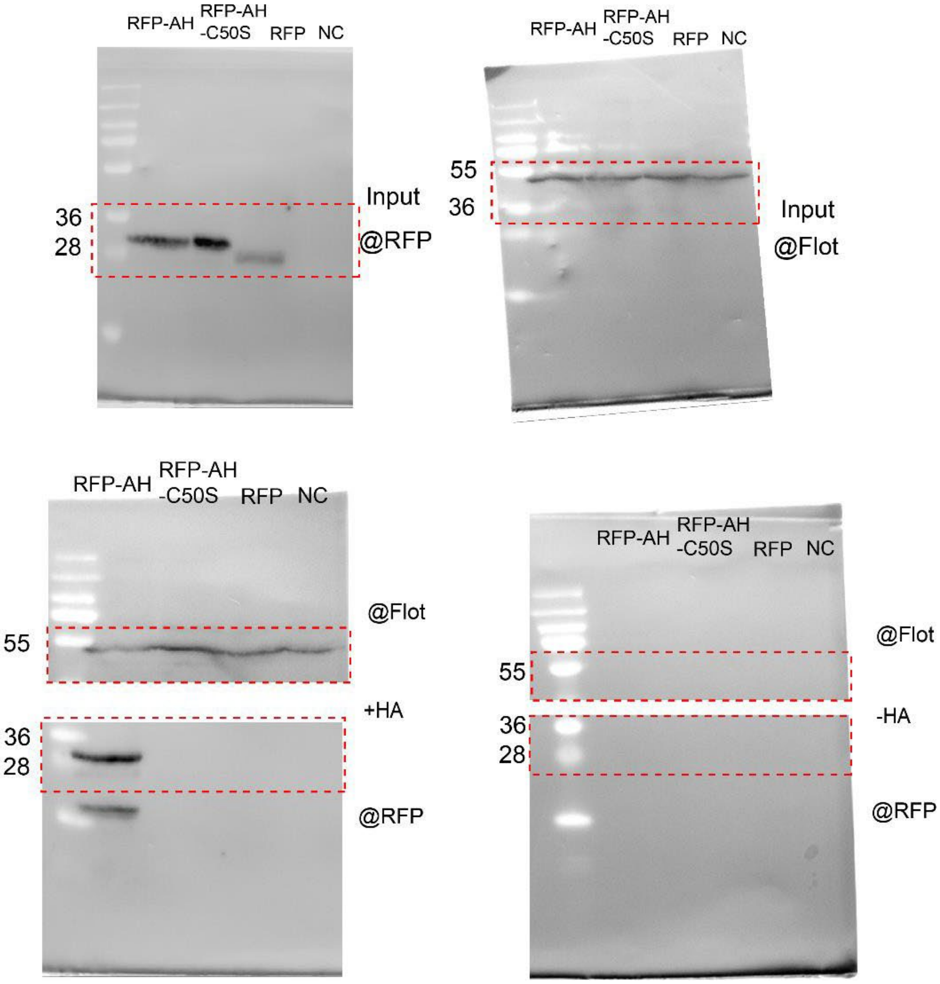

Full scan Figure 4

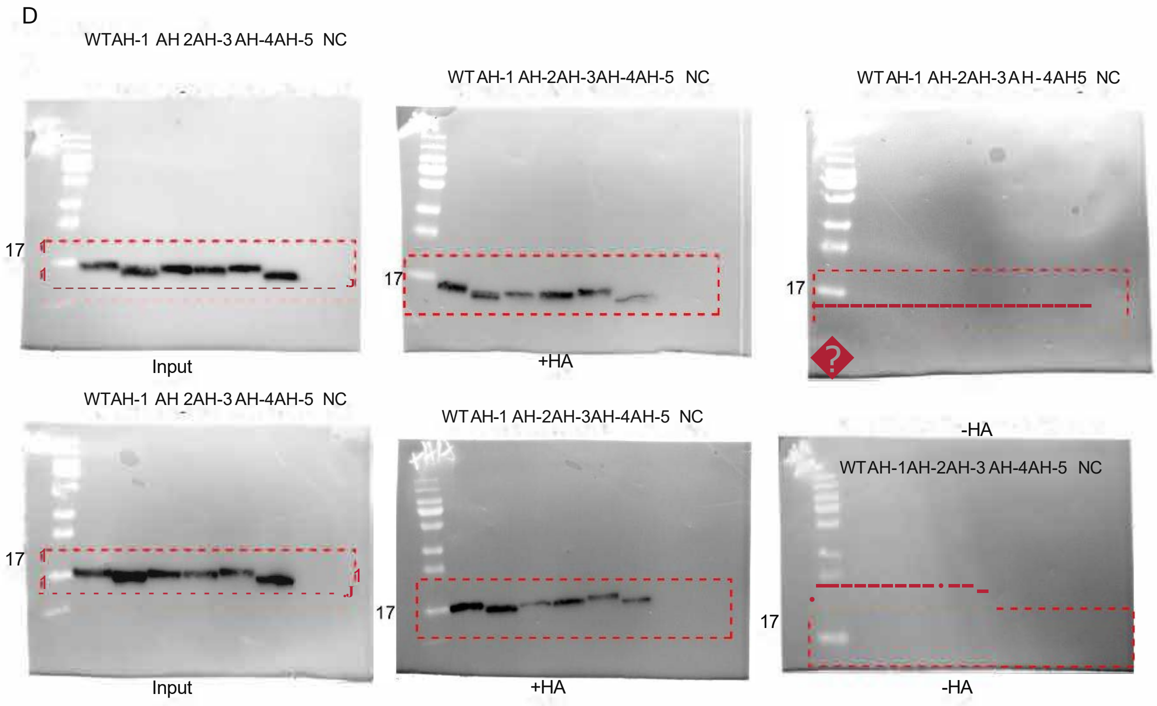

FULL SCAN Figure 5

C

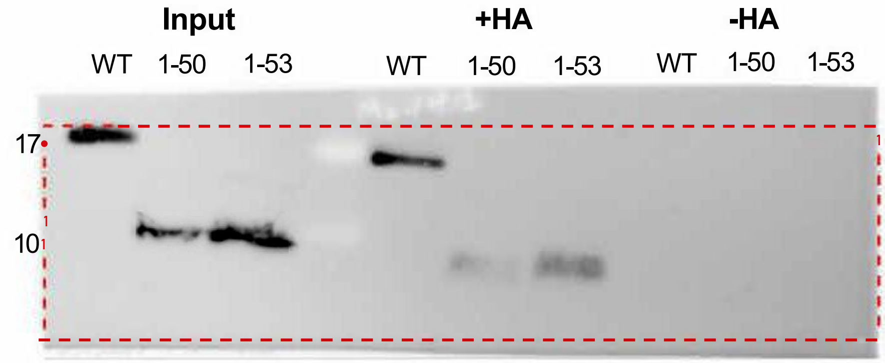

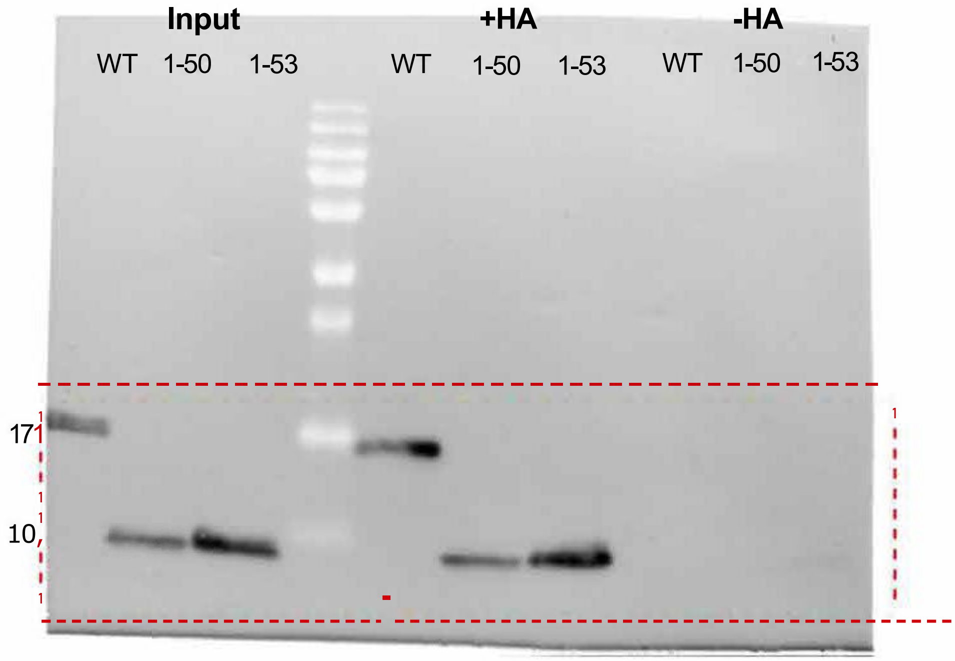

FULL SCAN Figure 6

C

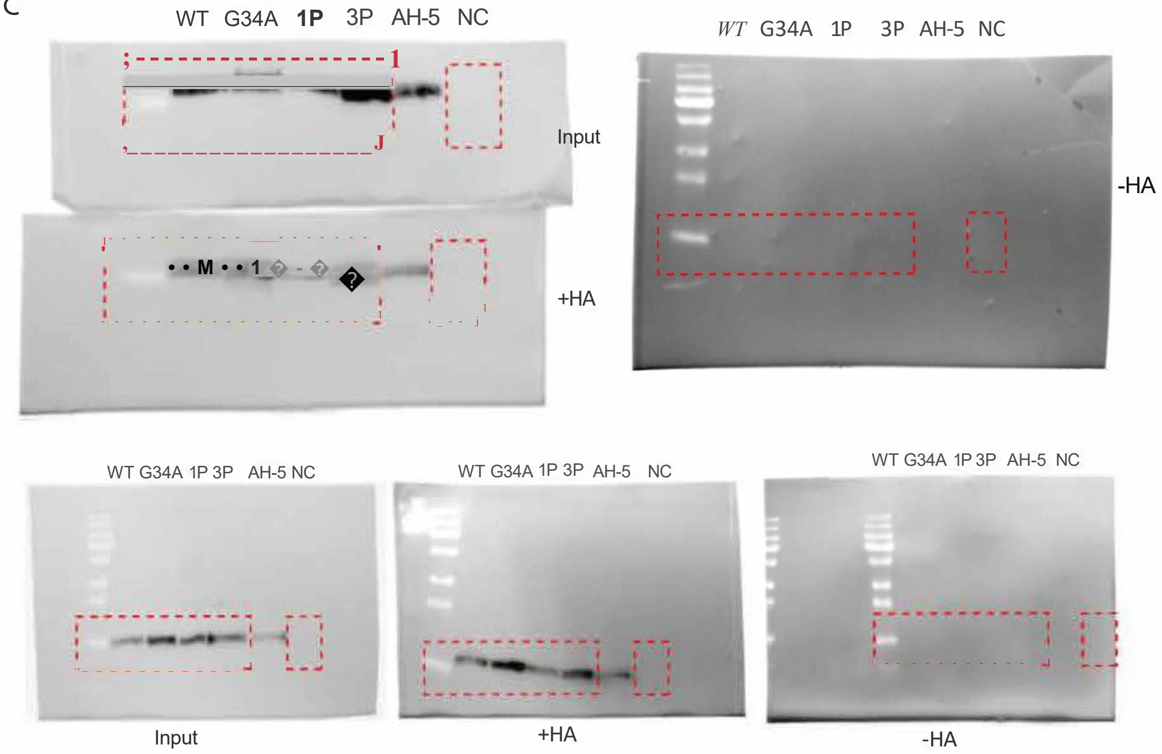

FULL SCAN Figure 7

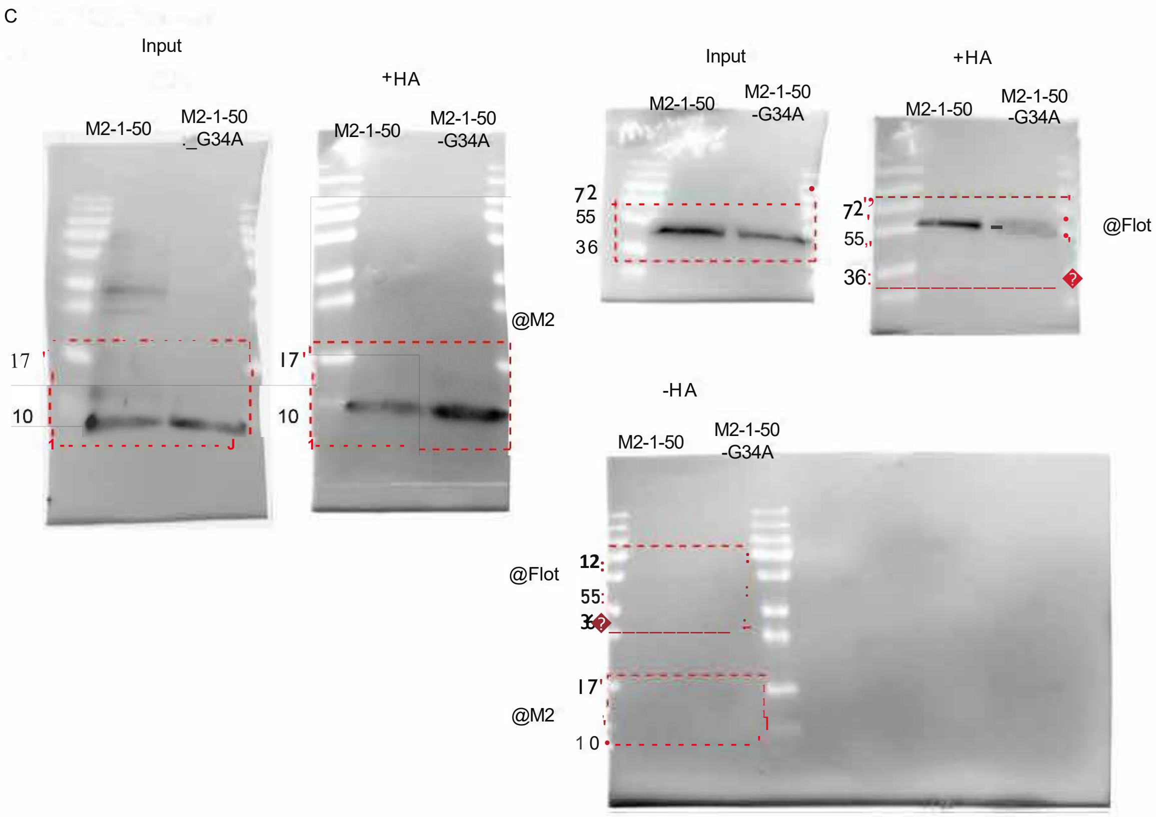

FULL SCAN Figure 8

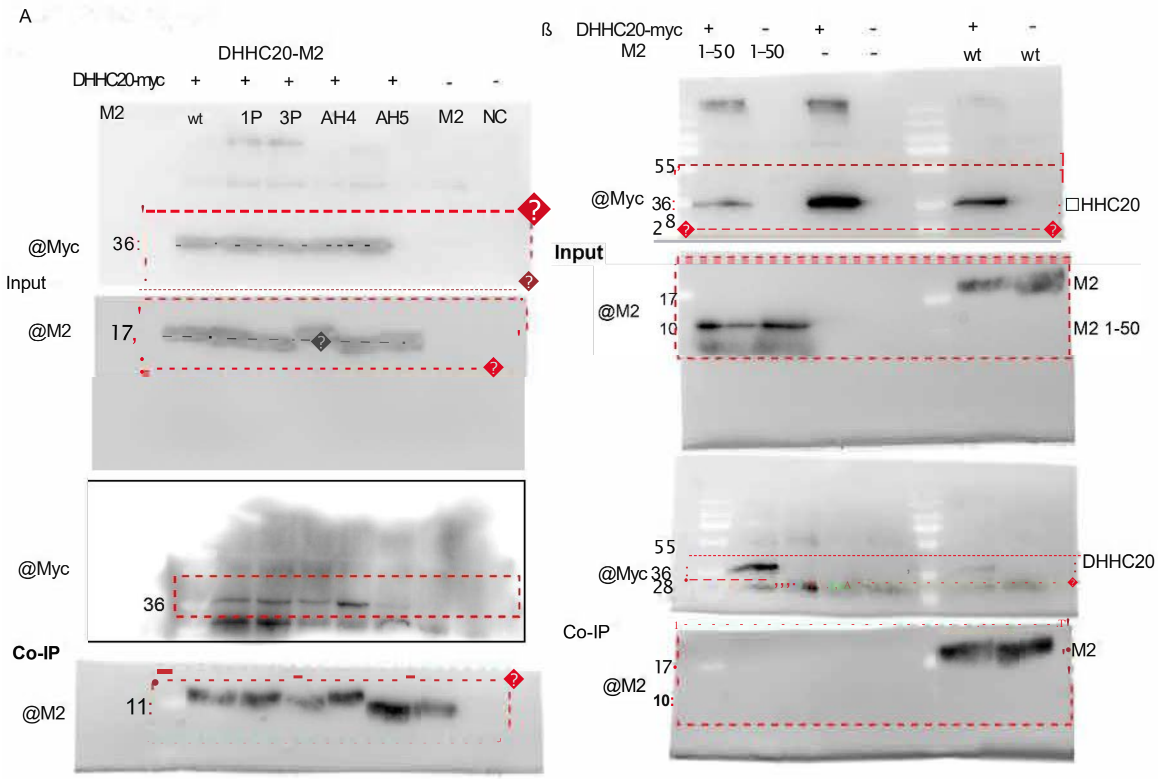

FULL SCAN Figure 8-2

FULL SCAN Figure 8
C

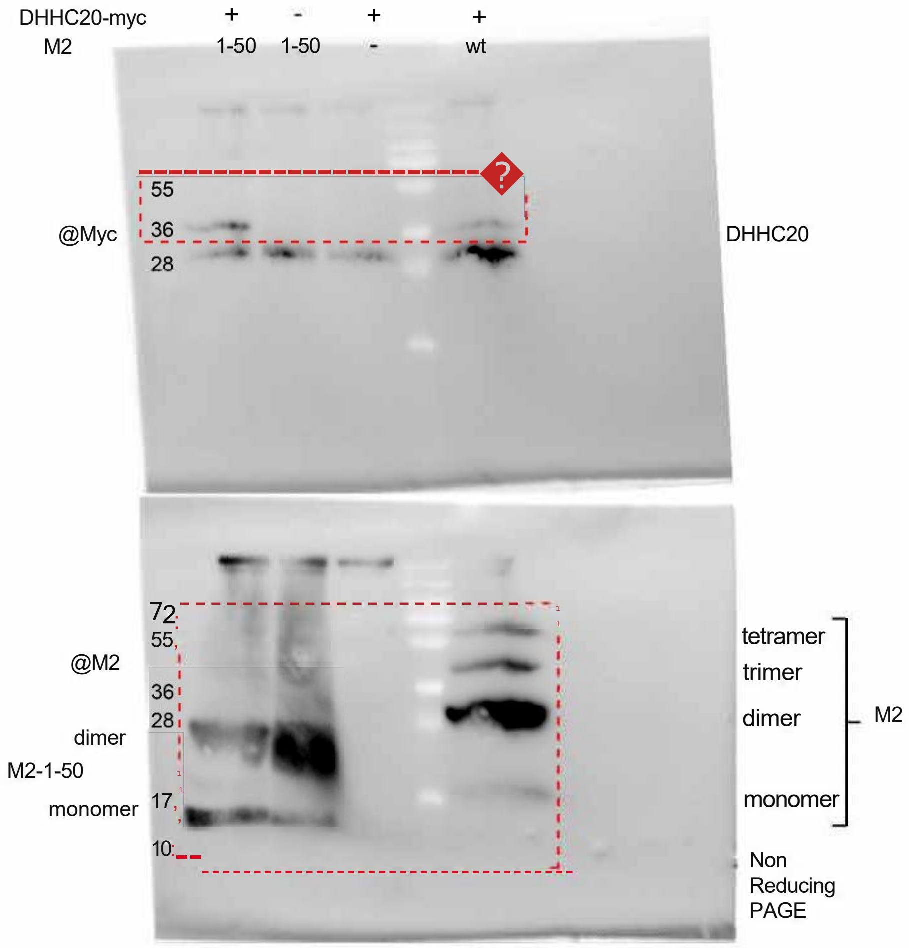

Full scan supplementary

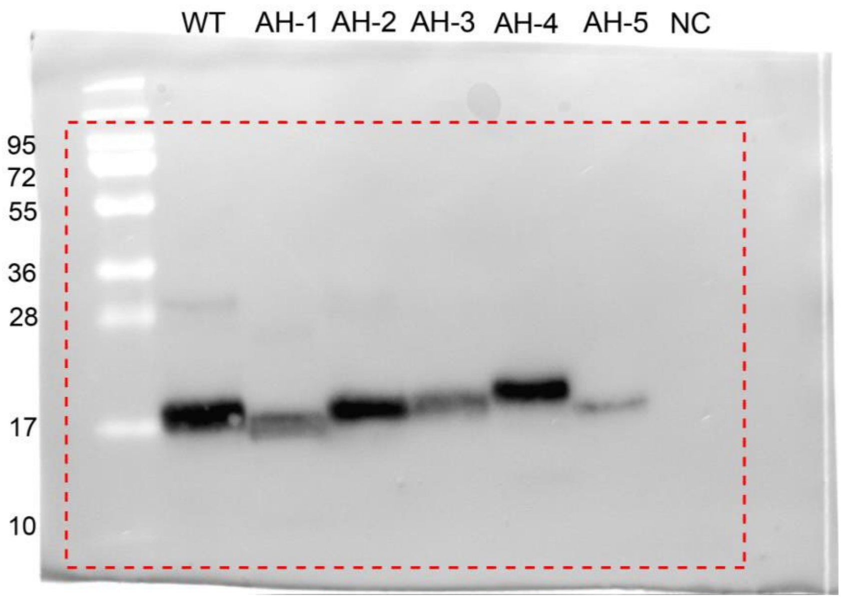

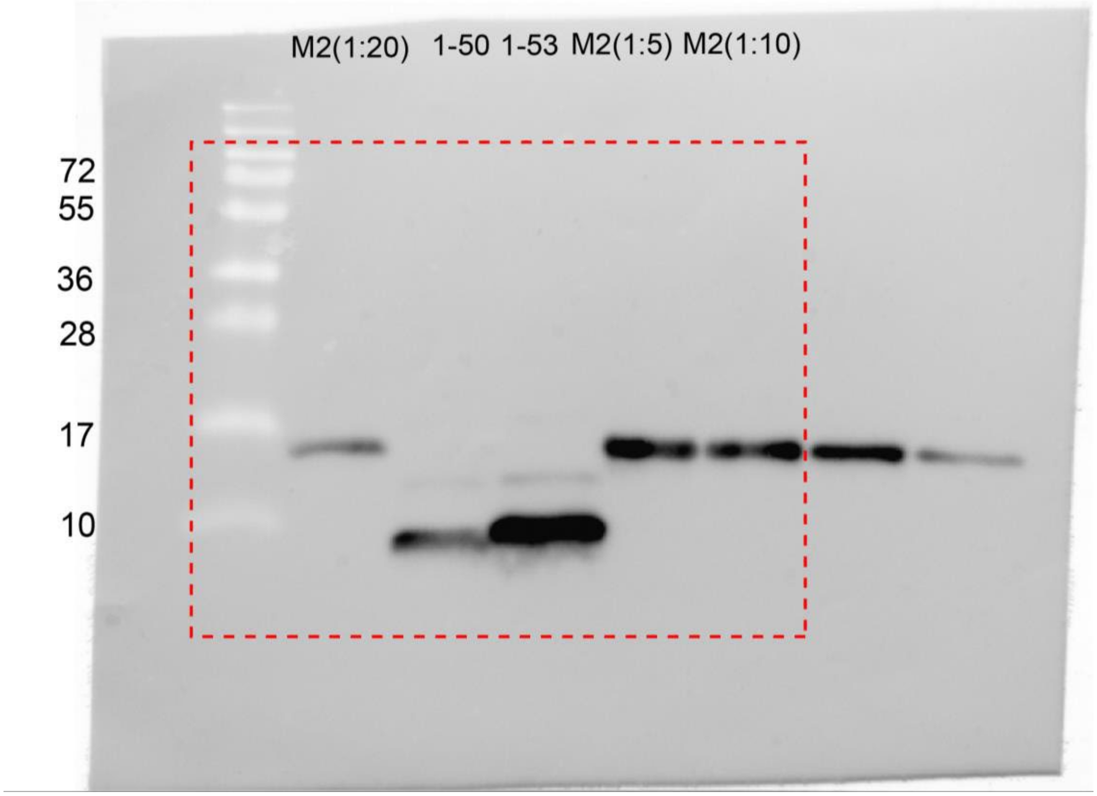

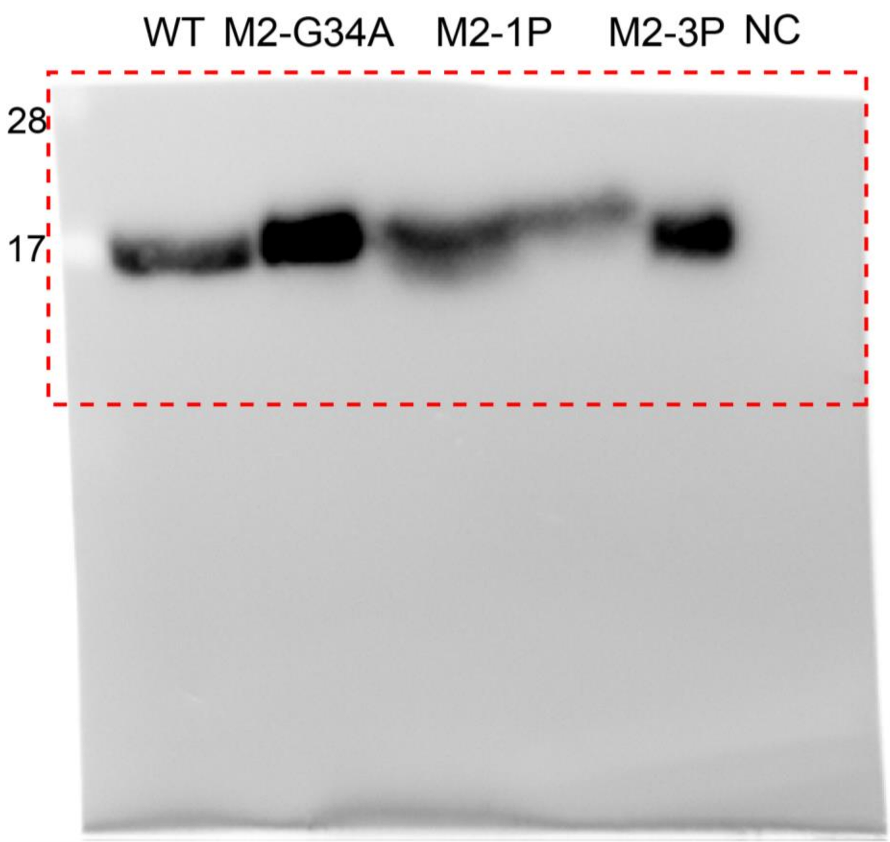

WT
M2-G34A
M2-1P
M2-3P
NC
28
17
